# Ablating UNG activity in a mouse model inhibits colorectal cancer growth by increasing tumor immunogenicity

**DOI:** 10.1172/jci.insight.184435

**Published:** 2025-07-15

**Authors:** Eric S. Christenson, Brandon E. Smith, Thanh J. Nguyen, Alens Valentin, Soren Charmsaz, Nicole E. Gross, Sarah M. Shin, Alexei Hernandez, Won Jin Ho, Srinivasan Yegnasubramanian, James T. Stivers

**Affiliations:** 1 Department of Oncology, Sidney Kimmel Comprehensive Cancer Center at Johns Hopkins,; 2 Cancer Convergence Institute at Johns Hopkins, and; 3 Department of Pharmacology and Molecular Sciences, Johns Hopkins University School of Medicine, Baltimore, Maryland, USA.

**Keywords:** Genetics, Immunology, Oncology, Cancer immunotherapy, Colorectal cancer, DNA repair

## Abstract

Uracil DNA glycosylase (UNG) excises uracil and 5-fluorouracil bases from DNA and is implicated in fluorodeoxyuridine (FdU) resistance. Here we explore the effects of inhibiting UNG activity, or depleting the UNG protein, in 2 mouse syngeneic models for colorectal cancer. Overexpressing the small UNG inhibitor protein (UGI) in mismatch repair–deficient (MMR-deficient) MC38 cells injected into C57BL/6J mice delayed tumor growth and prolonged survival when combined with FdU. Combining UNG inhibition with FdU numerically increased CD4^+^ T lymphocytes and B cells compared with FdU or UNG inhibition alone, suggesting an immune component to the effects. In contrast, shRNA depletion of UNG in the absence of FdU treatment resulted in 70% of mice clearing their tumors, and a 3-fold increase in overall survival compared with FdU. Analysis of MC38 tumor–infiltrating immune cells showed UNG depletion increased monocyte and dendritic cell populations, with CD8^+^ T cells also numerically increased. shRNA depletion of UNG in MMR-proficient CT-26 cells injected into BALB/c mice produced minimal benefit; the addition of anti–PD-1 antibody synergized with UNG depletion to increase survival. Cytotoxic T cell depletion abolished the benefits of UNG depletion in both models. These findings suggest UNG inhibition and/or depletion could enhance antitumor immune responses in humans.

## Abstract 2

We report on the observation that UNG depletion leads to improved survival in murine syngeneic colorectal cancer models through enhanced tumor immunogenicity.

## Introduction

**Figure 1:**
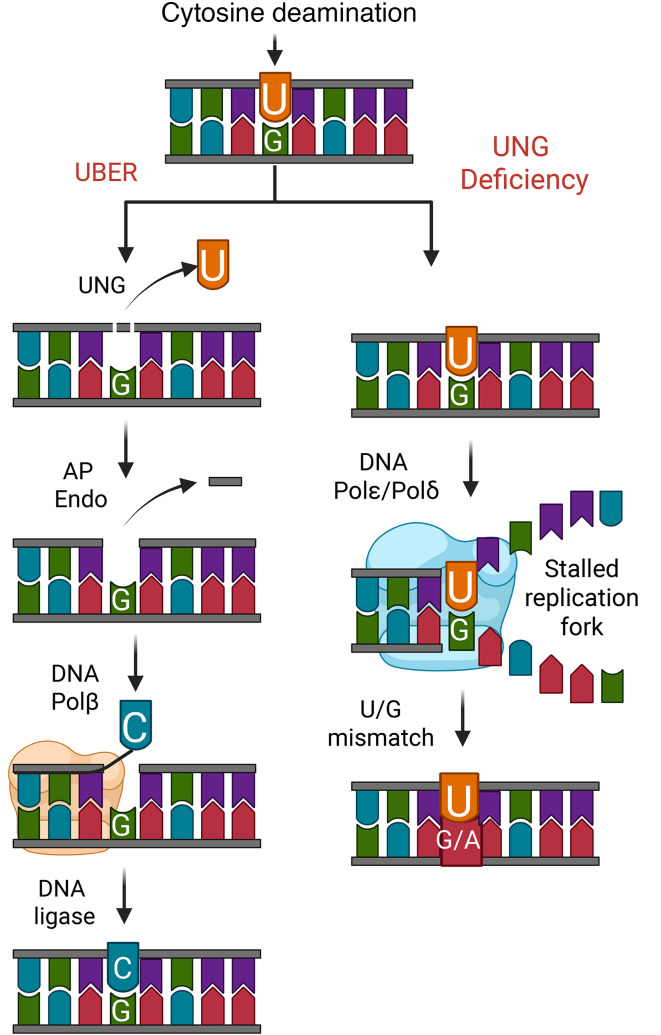
Loss of uracil DNA glycosylase (UNG) activity leads to persistence of uracil (U) and 5-fluorouracil (FU) bases in DNA. The uracil base excision repair pathway (UBER, left) efficiently excises U and FU (not shown) bases from DNA irrespective of whether the base pairs involve A or G. After the multiple-step pathway is complete, the excised nucleotide is replaced with either dTMP or dCMP using the coding information of the complementary strand base. In the absence of UNG activity (right), U or FU residues persist, leading to transition mutations after the DNA replication machinery encounters the lesion (U/G → T/A). U and FU lesions located on the template strand have been implicated in stalled DNA replication forks, and due to increased wobble base pairing, increase the misincorporation of guanine opposite to the U or FU base. Created with Biorender.

Uracil is a predominant noncanonical base found in genomic DNA that arises primarily from deoxyuridine triphosphate (dUTP) incorporation opposite to adenine by replicative DNA polymerases, producing nonmutagenic U/A base pairs (1). To a lesser extent, uracil also arises from spontaneous and enzymatic deamination of genomic cytosine bases, leading to mutagenic U/G mispairs (Figure 1). Genomic uracil arising from either pathway is efficiently excised by the uracil base excision repair (UBER) enzyme uracil DNA glycosylase (UNG) (2). The hijacking of normal thymidine biosynthesis by fluoropyrimidine (FP) drugs such as 5-fluorouracil (FU), 5-fluorodeoxyuridine (FdU), or the thymidylate synthase inhibitor pemetrexed, increases the levels of cellular dUTP and/or 5-fluorodeoxyuridine triphosphate (F-dUTP), all of which can be efficiently incorporated into DNA and excised by UNG (Figure 1) (3). In the absence of UNG, the persistence of U or FU in the template strand after 1 round of DNA replication potentiates fork arrest at the subsequent round of DNA replication and increases the toxicity of FdU in many human cancer cell lines (3–6). Thus, increased levels of U and FU in DNA are strongly associated with the therapeutic benefits of FP cancer therapy, in addition to alternative mechanisms involving incorporation of FU into RNA (7).

We and others have described how UNG protein depletion, or inhibition of its catalytic activity using the small UNG inhibitor protein (UGI), can significantly increase the potency of FdU or pemetrexed in human cancer cell lines (3–6, 8, 9). The Gerson group reported that a short-hairpin RNA (shRNA) targeting human *UNG* led to sensitization of p53-mutant cancer cells to FdU (5), and that depletion of UNG in a mouse xenograft model of lung cancer reduced tumor growth in combination with pemetrexed treatment (10). Lentiviral transduction of doxycycline-inducible UGI into 11 human colorectal cancer (CRC) cell lines showed a 6- to 70-fold increase in FdU potency for 6 of the cell lines, suggesting that therapeutic potential exists for small-molecule targeting of hUNG (3). Despite the general agreement between the UNG depletion and inhibition results in cell lines, the fact that UNG uses its 90–amino acid noncatalytic N-terminal domain (NTD) to interact with replication protein A (RPA) and proliferating cell nuclear antigen (PCNA), raises the possibility that loss of the protein-protein interactions in the depletion approach could be more detrimental to cancer cell growth than inhibition alone in some contexts, including in vivo studies (11–16). Indeed, recent findings from several groups have established that the prereplicative excision of uracil by UNG depends on its interaction with both PCNA and RPA and that removal of uracil in the template strand precipitates fork arrest and reversal, followed by repair and replication restart (4, 14).

There has been substantial interest in taking advantage of the defective DNA damage repair (DDR) phenotypes of some cancers (17), or using drugs that target DNA replication (18, 19), to increase the immunogenicity of tumors. Such strategies are typically based on paradigms of increasing the tumor mutational burden (TMB) and resulting tumor-associated neoantigens (20), or increasing type I IFN expression through nucleic acid–driven cGAS/STING activation (21). Both approaches are expected to be complementary with immune checkpoint blockade (ICB). The strong association of FdU and UNG with replication fork arrest suggests that nucleic acid–driven immune-potentiating effects might be expected in this context. This view, while speculative, is supported by the observation that replication-targeting agents result in rapid immune-potentiating outcomes, while increases in TMB require more time. Thus, in vivo studies with immunocompetent mice focusing on UNG inhibition/depletion in the context FdU treatment are of interest in testing these mechanisms.

In this study, we explore the therapeutic effects of FdU chemotherapy combined with UNG inhibition or depletion in 2 different syngeneic mouse models with different DDR phenotypes. When MC38 mismatch repair–deficient (MMR-deficient) CRC cells were inoculated into C57BL/6J mice and UGI expression was induced to inhibit UNG activity, delayed tumor growth and prolonged survival were observed only when combined with FdU treatment. Surprisingly, shRNA depletion of UNG (shRNA^UNG^) in the same MC38-C57BL/6J mouse model resulted in 70% of the mice clearing their tumors, and the addition of FdU had no further effect. In contrast, when MMR-proficient CT-26 colorectal tumor cells were injected into BALB/c mice, shRNA depletion of UNG showed no therapeutic benefit alone, or when combined with FdU, but a beneficial effect on tumor growth and survival was observed when UNG depletion was combined with anti–PD-1 (aPD1) antibody therapy. These combined findings suggest that UNG depletion alone can potentiate immunogenicity of tumors in a DDR-deficient mouse even in the absence of chemotherapy (i.e., MC38 cells in C57BL/6J mice), and that increased tumor immunogenicity is a key in vivo manifestation of UNG depletion.

## Results

### Inhibition and shRNA depletion of UNG in MC38 and CT-26 mouse CRC cells.

**Figure 2:**
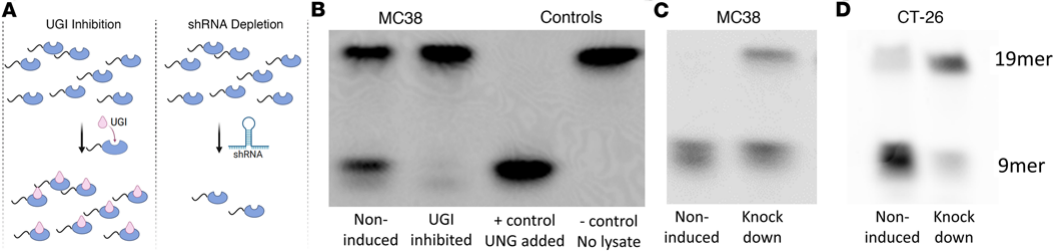
Two approaches for disrupting uracil DNA glycosylase (UNG) activity. (**A**) Two parallel approaches were used to understand whether inhibition of UNG activity or depletion of the entire enzyme molecule produced similar effects in syngeneic mouse models of colorectal cancer. Inhibition of UNG catalytic activity was achieved through inducible expression of the potent small UNG inhibitor (UGI) protein in the MC38 murine colon cancer cell line using lentiviral transduction methods. This maintains scaffolding functions of the protein and abrogates enzymatic activity. Depletion of the total UNG protein was achieved through doxycycline-inducible expression of *UNG*-specific shRNA (shRNA^UNG^ sequence A) using lentiviral transduction methods. This inhibits both scaffolding and enzymatic functions. Depletion may not be equivalent to inhibition because the UNG N-terminal domain (NTD) remains intact in the context of UGI inhibition, which could still allow UNG to localize to replication forks through its known protein-protein interactions (see text). (**B**) To establish the inducible inhibition by UGI, we collected cell extracts from induced and noninduced MC38 cell cultures and measured the UNG activity using a 5′-FAM–labeled 19mer DNA substrate containing a single uracil. Excision of uracil from this substrate results in a 9mer 5′-FAM–labeled product that can be resolved from the substrate by denaturing polyacrylamide gel electrophoresis. Greater than 97% inhibition of UNG activity was achieved through induction of UGI. Controls included the addition of purified UNG enzyme to the substrate in the absence of extract, and a no-extract negative control. (**C**) The same activity assay was used to test the efficacy of inducible shRNA^UNG^ in MC38 cells (70% knockdown), and (**D**) CT-26 cells (90% knockdown). Two biological replicates of each UNG activity experiment were performed.

We previously reported that inhibition of the catalytic activity of human UNG using the UGI protein increased the potency of FdU in over 50% of the human CRC lines that were tested (3). Similar effects have also been reported using shRNA knockdown of the UNG protein (4). As noted above, shRNA depletion may not always be equivalent to inhibition because the UNG NTD can still bind to RPA and PCNA in the context of UNG inhibition, allowing UNG to localize at replication forks and serve its scaffolding function (Figure 2A) (13).

To compare the effects of UNG inhibition and shRNA depletion in MC38 and CT-26 mouse CRC cells, we first assessed the extent of inhibition and depletion using cell extracts and a previously described UNG uracil excision activity assay (Figure 2, B–D) (22). This assay measures the glycosylase activity through the use of a 5′ fluorescein–labeled (5′-FAM–labeled) ssDNA that contains a single deoxyuridine residue. Excision of the uracil by UNG produces a 9mer DNA fragment that is diagnostic and highly specific for UNG activity. Specificity is confirmed by addition of purified UGI to the extract, which results in complete inhibition of the UNG activity in the extract, but not other glycosylases (23). These activity measurements revealed that UGI induction in MC38 cells resulted in complete inhibition of UNG activity, while shRNA depletion in MC38 and CT-26 cells resulted in 70% and 90% inhibition of the catalytic activity, respectively (Figure 2, B–D).

We also determined that UNG inhibition or depletion had no effect on the in vitro growth rate of MC38 or CT-26 cells (Supplemental Figure 1; supplemental material available online with this article; https://doi.org/10.1172/jci.insight.184435DS1), and therefore, any observed differences in tumor growth in mice cannot arise from these effects. Furthermore, unlike many human CRC cells previously studied (22), neither the MC38 nor CT-26 cells showed enhanced FdU cytotoxicity when UNG was inhibited or depleted (Supplemental Figure 2). Indeed, in vitro dose-response studies with FdU showed that MC38 cells were protected against FdU cytotoxicity when UGI was induced. Similarly increased resistance to FdU was previously observed for a human cell line (HCC2998) when UGI expression was induced, but the detailed basis for this protective response in some cell lines is not known. Importantly, these in vitro findings indicate that any beneficial therapeutic effect of UNG inhibition/depletion in syngeneic mice could not reasonably be attributed to increased tumor cell toxicity of FdU.

### Antitumor effects of UNG inhibition and FdU chemotherapy in C57BL/6J mice.

**Figure 3:**
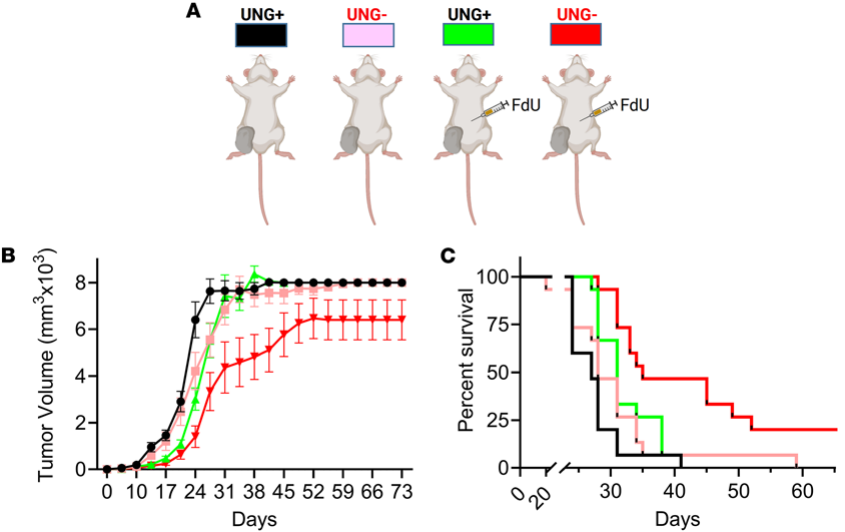
MC38 tumor growth and mouse survival in a syngeneic colon tumor model using FdU alone or in combination with UNG inhibition with UGI. We introduced the cDNA sequence of UGI into a CW57.1 doxycycline-inducible lentiviral expression plasmid and introduced the expression construct into MC38 cells. After confirming successful inducible inhibition of UNG in the transduced cell line (see Figure 2B), 2.5 × 10^5^ MC38 cells were injected into the right hind limb of female C57BL/6J mice. Overall, 60 mice were used in 2 independent experiments. In the first experiment, the mice were divided into 4 groups of 5 mice, and the second, 4 groups of 10 mice. The 4 experimental groups were (a) *UNG^+^*, empty vector with no UNG inhibition and no FdU treatment, (b) *UNG^–^*, UNG inhibition with no FdU treatment; 1, (c) FdU treatment with no UNG inhibition (*UNG^+^*/FdU) and, (d) combination of UNG inhibition and FdU treatment (*UNG^–^*/FdU). (**A**) FdU was administered by intraperitoneal injection using a 50 mg/kg daily dose on days 3 to 12 and UNG inhibition was induced with doxycycline starting on day 1. (**B**) Tumor volumes for the 4 experimental groups were measured on the indicated days. Measurements from the replicate experiments were pooled and plotted for statistical analysis (*n* = 15 for each group). Error bars for tumor growth are SEM. Tumor growth curves were compared using an unpaired 2-tailed *t* test between groups: *UNG^–^*/FdU vs. *UNG^+^*/FdU (*P* = 0.09) and *UNG^–^*/FdU vs. *UNG^–^* (*P* = 0.12). (**C**) Kaplan-Meier survival analysis for the 4 experimental groups (*n* = 15 per group). Statistical comparisons of survival between groups used the log-rank Mantel-Cox test: *UNG^–^*/FdU vs. *UNG^+^*/FdU (*P* value = 0.005 and *UNG^–^*/FdU vs. *UNG^–^* (*P* = 0.005).

To determine the impact of UNG inhibition on MC38 tumor growth and mouse survival, we introduced the cDNA sequence of UGI into a CW57.1 doxycycline-inducible lentiviral expression plasmid and introduced the expression construct into MC38 cells, which were injected into the right hind limb of female C57BL/6J mice (60 mice total in 2 independent experiments) (Figure 3). The mice were divided into 4 experimental groups (Figure 3A): (a) *UNG^+^*, no doxycycline induction of UGI (UNG proficient) with no FdU treatment; (b) *UNG^–^*, doxycycline induction of UGI (UNG inhibition) with no FdU treatment; (c) *UNG^+^*/FdU, no UNG inhibition and FdU treatment; and (d) *UNG^–^*/FdU, combination of UNG inhibition and FdU treatment. In all test groups, FdU was administered by intraperitoneal injection using a 50 mg/kg daily dose on days 3 to 12, and UNG inhibition was induced with doxycycline starting on day zero. Tumor volumes and survival for the 4 experimental groups were measured over 70 days. A trend toward slower tumor growth was observed for the combination *UNG^–^*/FdU group as compared with the groups with FdU or UNG inhibition alone (*UNG^–^*/FdU vs. *UNG^+^*/FdU, *P* = 0.09; *UNG^–^*/FdU vs. *UNG^–^*, *P* = 0.12). (Figure 3B). A Kaplan-Meier survival analysis indicated significant differences between the test groups using the log-rank Mantel-Cox test (Figure 3C: *UNG^–^*/FdU vs. *UNG^+^*/FdU, *P* = 0.005; *UNG^–^*/FdU vs. *UNG^–^*, *P* = 0.005).

### UNG inhibition changes the immune microenvironment of MC38 tumors in C57BL/6J mice.

**Figure 4:**
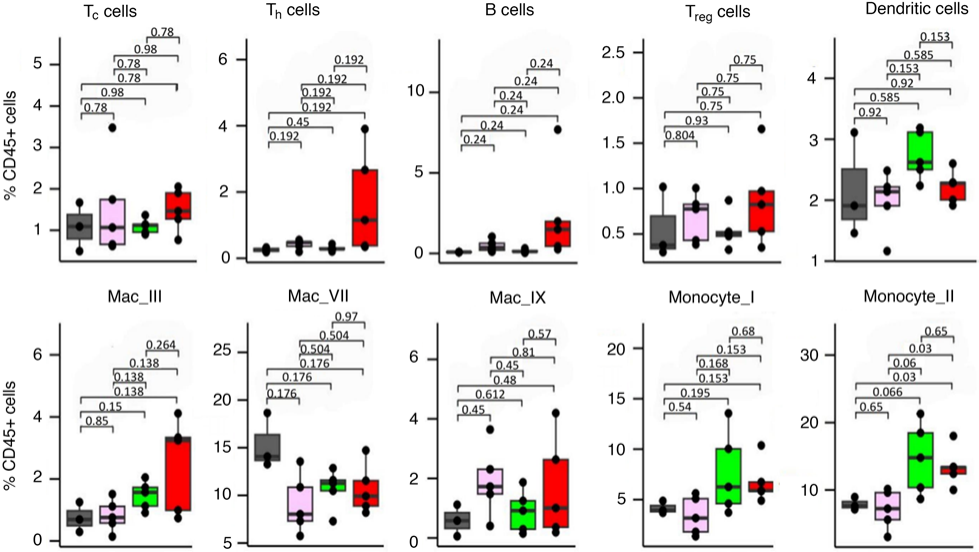
Measurement of the tumor microenvironment with CyTOF demonstrates inhibiting UNG catalytic activity with UGI in combination with FdU treatment alters the immune microenvironment. To determine the influence of UNG inhibition via UGI on the MC38 tumor microenvironment, 2.5 × 10^5^ MC38 cells containing the CW57.1 UGI guide were injected into the right hind limb of female C57BL/6J mice (20 total). Mice were divided into 4 groups of 10 mice; (a) Control, (b) UNG inhibition via doxycycline-induced UGI expression starting on day one, (c) floxuridine (FdU) treatment at 50 mg/kg/dose daily via intraperitoneal injection days 3 to 12, and (d) UNG inhibition combined with FdU treatment. On day 20, mice were sacrificed and tumors excised. Tumors were then homogenized and stained with a panel of 40 antibodies with the Fc region complexed with unique heavy metal isotopes. This demonstrated a numerical increase in CD4^+^ T lymphocytes and B cells in the combination treatment group. Selected cell subtypes are shown in the figure with an entire list and heatmap provided in Supplemental Figure 8. Data are shown as box-and-whisker plots, with the mean indicated by a horizontal line, middle quartiles represented by the box bounds, and range denoted by the whiskers. Significance calculated using pairwise 2-tailed *t* tests. *P* values were adjusted for multiple comparisons within each cell type using Benjamini-Hochberg. Tc cells, CD8^+^ T lymphocytes; Th cells, CD4^+^ T lymphocytes; Treg cells, regulatory T cells; Mac_III, CD206^–^CD11b^+^Ki67^+^ macrophages; Mac_VII, CD206^–^CD11b^+^Ki67^+^IAIE^+^ macrophages; Mac_IX, CD206^+^CD11b^+^Ki67^+^PD-L1^–^ macrophages; Monocyte_I, CD11b^+^Ly6G^+^ monocytes; Monocyte_II, CD11b^+^Ly6G^+^PD-L1^+^IAIE^+^ monocytes.

To determine the local immunological effects of UNG inhibition by UGI in the MC38-C57BL/6J mouse model, MC38 cells containing the UGI inducible expression construct were injected into the right hind limb of female C57BL/6J mice (4 groups of 10 mice as indicated in Figure 3A). Mice were sacrificed on day 20, and the tumors were excised, homogenized, and stained with a panel of 40 immune cell–specific antibodies tagged with heavy metal isotopes for analysis by mass cytometry by time of flight (CyTOF) (Figure 4 and Supplemental Figure 3) (24).

Several changes were notable within the myeloid compartment. UNG inhibition led to a trend toward increases in subpopulations of CD11b^+^F4/80^+^ macrophages that were positive for the MHC-II marker IAIE (Mac_I, Mac_IV; *P* = 0.156, *P* = 0.092). FdU treatment alone showed a trend toward enrichment for subsets of macrophages that were negative in expression of CD206 (Mac_II) and CD80^+^CD86^+^ (Mac_VI) (*P* = 0.18, *P* = 0.046). Also, FdU treatment also showed a trend toward increases in a Ly6C^+^ subset of monocytes positive for IAIE (Monocyte_II) (*P* = 0.066). Consistent with these changes within the myeloid compartment that suggest improved antigen presentation and T cell activation, increased CD8^+^ cytotoxic T (Tc) cells and CD4^+^ helper T (Th) cells were also observed in the combination arm, although these changes were not statistically significant.

### shRNA knockdown of UNG leads to clearance of MC38 tumors in C57BL/6J mice.

**Figure 5:**
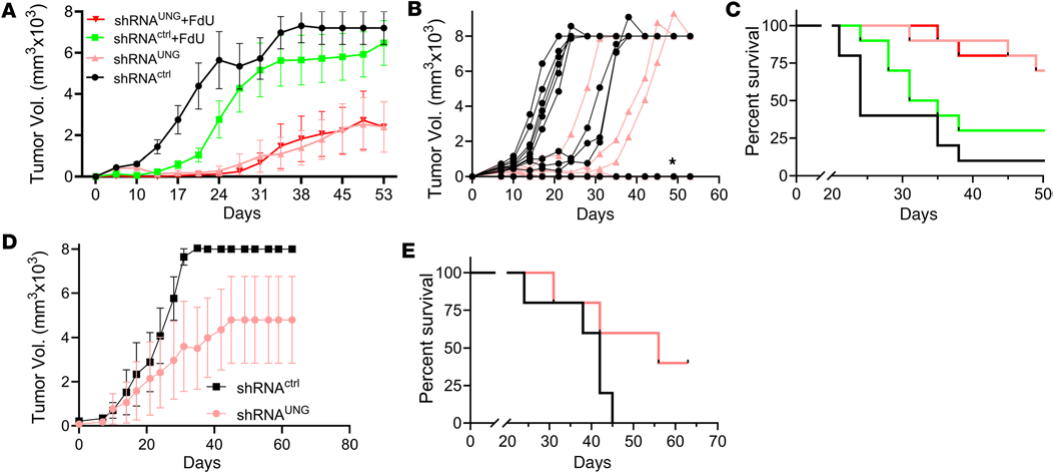
shRNA knockdown of UNG leads to clearance of MC38 tumors in C57BL/6J mice. To determine the antitumor effects of UNG protein depletion in the presence and absence of FdU treatment, we used 2 doxycycline-inducible shRNA constructs that targeted *UNG* (shRNA^UNG^ seq A, shRNA^UNG^ seq B), and a control shRNA (shRNA^ctrl^) that did not target any genomic mRNA sequence. After confirming successful delivery of the shRNA^UNG^ and shRNA^ctrl^ constructs, 2.5 × 10^5^ MC38 cells were injected into the right hind limb of female C57BL/6J mice. The experiment, which was performed twice, with each experiment consisting of 4 groups of 10 mice: (a) shRNA^ctrl^ without FdU treatment, (b) shRNA^ctrl^ with FdU treatment**,** (c) shRNA^UNG^ without FdU treatment, and (d) shRNA^UNG^ with FdU treatment. FdU treatment was performed in an identical fashion as described in the legend to Figure 3. (**A**) Tumor volumes for the 4 experimental groups were measured on the indicated days using shRNA^UNG^ sequence A. (**B**) Tumor volumes for 10 individual mice inoculated with shRNA^UNG^ sequence A and shRNA^ctrl^ in the presence and absence of FdU. The asterisk indicates the overlapping data for 7 mice receiving shRNA^UNG^ sequence A that had no palpable tumors. One mouse receiving the shRNA^ctrl^ also had no palpable tumors. Error bars are SEM. Tumor growth was compared using an unpaired 2-tailed *t* test between the experimental groups: shRNA^ctrl^ vs. shRNA^UNG^ sequence A (*P* < 0.0001), shRNA^ctrl^ + FdU treatment vs. shRNA^UNG^ (*P* = 0.003), and shRNA^ctrl^ + FdU vs. shRNA^UNG^ + FdU (*P* = 0.003). (**C**) Kaplan-Meier survival analysis for the 4 experimental groups. Seven out of 10 mice in both the shRNA^UNG^ (sequence A) and shRNA^UNG/FdU^ treatment groups had cleared their tumors 7 days after inoculation. Survival comparisons were made the log-rank Mantel-Cox test: shRNA^UNG^ vs. shRNA^ctrl^ (*P* = 0.0009), shRNA^ctrl^ + FdU treatment vs. shRNA^UNG^ (*P* = 0.013), and shRNA^ctrl^ + FdU vs. shRNA^UNG^ + FdU (*P* = 0.012). (**D** and **E**) Given the absence of an effect of FdU in the experiments in **A** and **B**, shRNA depletion of UNG was repeated in an additional experiment with the alternative shRNA guide shRNA^UNG^ sequence B in the absence of FdU (5 mice per group), showing similar results.

For comparison with the UGI inhibition results, we performed shRNA depletion of UNG in the MC38-C57BL/6J mouse model (Figure 5). A doxycycline-inducible shRNA construct that targeted *UNG* (shRNA^UNG^; sequence A), and a control shRNA (shRNA^ctrl^) that did not target any genomic mRNA sequence were introduced into MC38 cells (Supplemental Table 1). The transfected cells were injected into the right hind limb of female C57BL/6J mice in 2 independent experiments with 4 groups of 10 mice. Tumor volumes were measured on the indicated days for the 4 test groups (Figure 5A): (a) shRNA^ctrl^ without FdU treatment, (b) shRNA^ctrl^ with FdU treatment, (c) shRNA^UNG^ without FdU treatment, and (d) shRNA^UNG^ with FdU treatment. In contrast with UNG inhibition, UNG depletion alone showed a significant effect on tumor growth and the addition of FdU had no further benefit (Figure 5A). Seven out of 10 mice in both the shRNA^UNG^ and shRNA^UNG/FdU^ treatment groups had cleared their tumors 7 days after inoculation, as compared with only 1 mouse receiving the shRNA^ctrl^ (Figure 5B). The effects on tumor growth between the experimental groups were highly significant: shRNA^ctrl^ versus shRNA^UNG^ (*P* < 0.0001), shRNA^ctrl^ plus FdU treatment versus shRNA^UNG^ (*P* = 0.003), and shRNA^ctrl^ plus FdU versus shRNA^UNG^ plus FdU (*P* = 0.003). UNG depletion alone and in combination with FdU also produced a significant beneficial effect on survival (Figure 5C): shRNA^UNG^ versus shRNA^ctrl^ (*P* = 0.0009), shRNA^ctrl^ plus FdU treatment versus shRNA^UNG^ (*P* = 0.013), and shRNA^ctrl^ plus FdU versus shRNA^UNG^ plus FdU (*P* = 0.012).

In a control experiment, shRNA depletion of UNG was repeated using a different shRNA (shRNA^UNG^; sequence B) (Supplemental Table 1). Given the absence of an effect of FdU in the experiments in Figure 5, A and B, this control experiment was performed in the absence of FdU using 5 mice per experimental group (Figure 5, D and E). The results using this alternative shRNA were comparable to shRNA^UNG^ sequence A under the same condition. Thus, for all subsequent experiments described in the later figures and text, shRNA^UNG^ sequence A was used exclusively.

### shRNA knockdown of UNG increases immunological response to MC38 tumors in C57BL/6J mice.

We also used CyTOF to analyze the immune responses to shRNA^UNG^ and shRNA^ctrl^ in 20 female C57BL/6J mice (10 mice for each shRNA construct). On day 7, mice were sacrificed, and the tumors were excised, homogenized, and analyzed with CyTOF.

**Figure 6:**
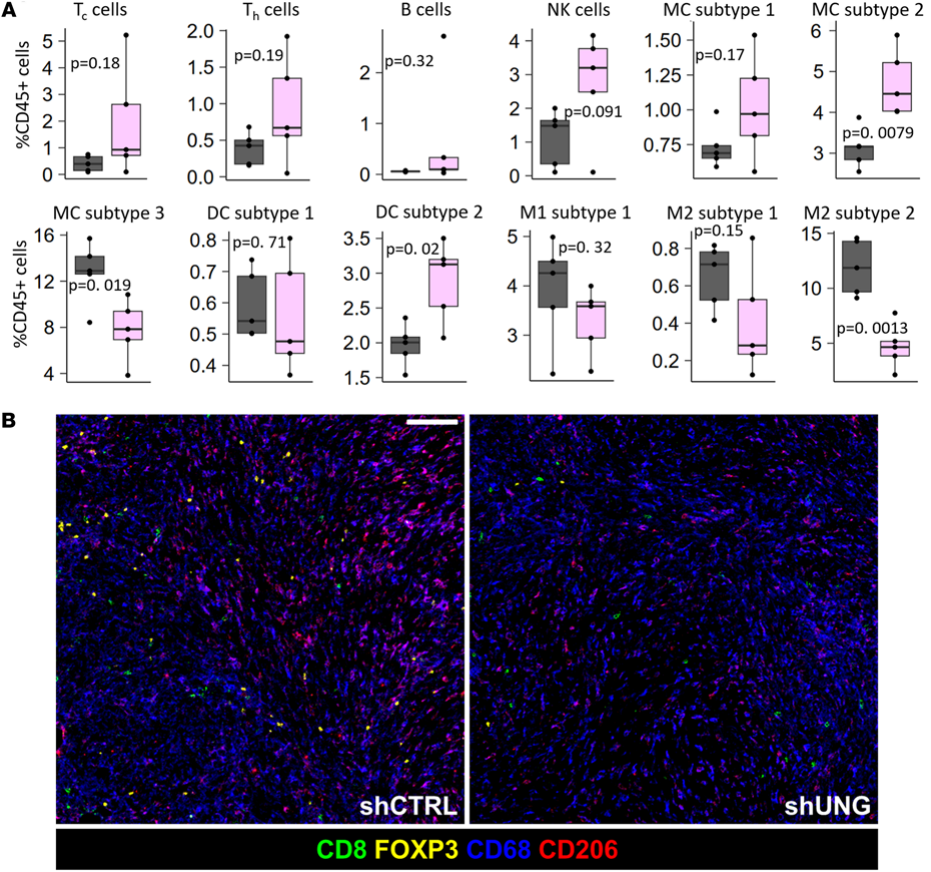
Determination of the influence of UNG depletion on the MC38 tumor microenvironment using CyTOF and IMC. (**A**) In the CyTOF experiment, 2.5 × 10^5^ MC38 cells expressing shRNA^UNG^ or shRNA^ctrl^ were injected into the right hind limb of female C57BL/6J mice (20 mice total: 10 mice for each experimental group). On day 7, the mice were sacrificed and the tumors were excised. Tumors were homogenized and stained with a panel of 40 antibodies tagged with heavy metal isotopes for analysis by CyTOF. A Kruskal-Wallis analysis demonstrated statistically significant changes in monocyte, DC, and macrophage subpopulations. Selected cell subtypes are shown in the figure. The entire list and a heatmap plot of the data is provided in Supplemental Figures 7 and 9. Data are shown as box-and-whisker plots, with the mean indicated by a horizontal line, middle quartiles represented by the box bounds, and range denoted by the whiskers. Tc cells, CD8^+^ T lymphocytes; Th cells, CD4^+^ T lymphocytes; NK cells, natural kill cells; DC subtype 1, CD11B^+^IAIE^+^CD80^+^CD11C^+^CD40^+^; DC subtype 2, CD11B^+^IAIE^+^CD80^–^CD11C^+^CD40^+^; Mac_I, CD206^–^CD11B^+^IAIE^–^Ki67^–^CD40^–^Ly6C^–^ macrophages; Mac_III, CD206^+^PD-L1^+^Ki67^–^CD40^+^ macrophages; Mac_IV, CD206^+^PD-L1^+^CD40^–^ macrophages; Monocyte_I, Ly6C^+^PD-L1^–^IAIE^+^CD40^–^Ki67^–^CD206^–^ monocytes; Monocyte_II, Ly6C^+^PD-L1^+^IAIE^–^CD40^–^Ki67^–^CD206^–^ monocytes; Monocyte_III, Ly6C^+^PD-L1^+^IAIE^+^CD40^–^Ki67^–^CD206^–^ monocytes. (**B**) Shows representative images from the IMC experiment demonstrating lower levels of intratumoral Tregs and higher levels of cytotoxic T cells in the UNG-depleted tumors. Scale bar: 100 μm. IMC subpopulation densities are shown in Supplemental Figure 10.

Statistically significant increases in monocyte (MC) subtype 2 (subtype of CD11b^+^Ly6C^+^ monocytes [PD-L1^hi^]) and CD11c^+^ dendritic cells (DC subtype 2 [IAIE^hi^CD40^hi^PD-L1^hi^CD80^lo^]), were observed between the shRNA^UNG^ and shRNA^ctrl^ groups (*P* = 0.0079 and *P* = 0.02, respectively). We also observed decreases in “M2 subtype 2,” which have antiinflammatory M2-like cell surface markers (IAIE^–^CD40^–^CD206^+/int^) (*P* = 0.0013) (Figure 6). As observed with UGI inhibition, higher mean numbers of Tc, Th, and B cells were observed in the shRNA^UNG^ group, but the effects were not statistically significant (*P* > 0.05).

We also used imaging mass cytometry (IMC) to analyze the intratumoral immune microenvironment of MC38 tumors when shRNA^UNG^ and shRNA^ctrl^ were expressed in female C57BL/6J mice (4 mice for each shRNA construct). It should be noted that quantitative agreement between CyTOF and IMC measurements may not be observed because the CyTOF analysis is referenced to live cells and reports on intratumoral and peritumoral cell populations, whereas IMC is referenced to CD45^+^ cells and reports only intratumoral populations. Nevertheless, the approaches provide a complementary picture of immune cell populations responding to the tumor phenotype. For the IMC analysis, mice were sacrificed on day 7, tumors excised, fixed in formalin, cut, and placed onto slides for analysis.

IMC identified significantly higher numbers of proliferative T cells in MC38 tumors expressing shRNA^UNG^ as compared with shRNA^ctrl^ (*P* = 0.014), indicating a strong general T cell response to UNG depletion. Consistent with the CyTOF findings, there were higher mean numbers of Tc lymphocytes, although these did not reach statistical significance (*P* = 0.25). The IMC analysis detected higher numbers of M1 macrophages (CD68^+^CD11c^+^) in the setting of UNG depletion (*P* = 0.021) (Supplemental Figure 4). IMC also detected lower numbers of granulocytes and Tregs in the UNG depletion condition, although these results did not reach high statistical significance (*P* = 0.14 and *P* = 0.21, respectively). (Granulocytes and Tregs were not analyzed in the CyTOF experiment.) Thus, IMC identified changes in the intratumoral compartment that trended toward higher numbers of Tc lymphocytes and M1 macrophages. There were also trends toward reduced numbers of Tregs and granulocytes, suggesting that depletion of UNG produces antitumor effects in the tumor microenvironment (representative image, Figure 6B).

### Depletion of CD8^+^ T lymphocytes ablates the tumor growth and survival benefits of UNG knockdown in the MC38 murine model.

**Figure 7:**
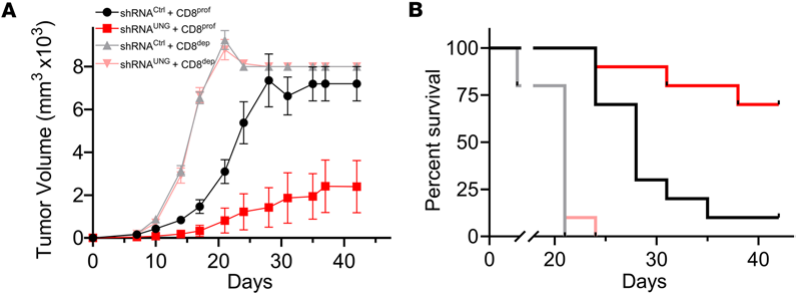
Depletion of CD8^+^ T lymphocytes ablates the tumor growth and survival benefits of UNG depletion in MC38 tumor–bearing mice in the absence of FdU. C57BL/6J mice were treated with anti-CD8 depletion antibodies twice weekly to interrogate the contribution of CD8^+^ lymphocytes to the tumor clearance observed with UNG knockdown. Female C57BL/6J mice were injected with 2.5 × 10^5^ MC38 cells containing either shRNA^UNG^ or shRNA^ctrl^. The CD8^+^ cell depletion experiment was performed twice with 5 mice per experimental group. These panels show the combined data from both experiments: (a) shRNA^ctrl^ without CD8^+^ cell depletion (shRNA^ctrl^ CD8^proficient^), (b) shRNA^ctrl^ with CD8^+^ cell depletion (shRNA^ctrl^ CD8^depleted^), (c) shRNA^UNG^ without CD8^+^ cell depletion (shRNA^UNG^ CD8^proficient^), and (d) shRNA^UNG^ with CD8^+^ cell depletion (shRNA^UNG^ CD8^depleted^). (**A**) Tumor volumes for the 4 experimental groups were measured on the indicated days. Error bars for tumor growth are SEM. Tumor growth curves were compared using an unpaired 2-tailed *t* test. For shRNA^UNG^ CD8^proficient^ tumors, there was significantly reduced tumor growth as compared with both CD8^depleted^ groups (*P* = 0.007). (**B**) Kaplan-Meier survival analysis for the 4 experimental groups. For shRNA^UNG^ CD8^proficient^ tumors, there was significantly improved survival in the UNG-depleted group as compared with both CD8^depleted^ groups (*P* = 0.003, log-rank Mantel-Cox test). Seven of the mice in the shRNA^UNG^ CD8^proficient^ group cleared their tumors.

Since the CyTOF analyses of the UGI/FdU combination and single-agent shRNA^UNG^ regimes suggested changes in the tumor microenvironment that could promote the action of CD8^+^ Tc cells, we performed a depletion experiment where C57BL/6J mice were treated with anti-CD8 depletion antibodies twice weekly (Figure 7). Since there was no beneficial effect of FdU in the context of expressing shRNA^UNG^ in MC38 tumors (Figure 5), the depletion of CD8^+^ Tc cells was performed without administering FdU. Accordingly, the test groups were (a) shRNA^ctrl^ without CD8^+^ cell depletion (shRNA^ctrl^ CD8^proficient^), (b) shRNA^ctrl^ with CD8^+^ cell depletion (shRNA^ctrl^ CD8^depleted^), (c) shRNA^UNG^ without CD8^+^ cell depletion (shRNA^UNG^ CD8^proficient^), and (d) shRNA^UNG^ with CD8^+^ cell depletion (shRNA^UNG^ CD8^depleted^). The experiment was performed twice with 5 mice per experimental group and the combined data from both experiments are shown in Figure 5. Consistent with a key role of CD8^+^ T cells in the observed antitumor effects of the UNG knockdown, tumor volumes for the shRNA^UNG^ CD8^proficient^ experimental group were significantly reduced as compared with the shRNA^UNG^ CD8^depleted^ group (*P* = 0.007) (Figure 5A). Indeed, in the absence of the CD8^+^ cell response, the shRNA^UNG^ CD8^depleted^ group fared no better than the shRNA^ctrl^ CD8^depleted^ group. Consistent with the effects on tumor growth, the CD8^proficient^ tumors with UNG depletion showed significantly improved survival as compared with the CD8^depleted^ groups (*P* = 0.003). Indeed, 7 of the mice in the shRNA^UNG^ CD8^proficient^ group cleared their tumors, while only 1 mouse in the shRNA^ctrl^ CD8^proficient^ group showed clearance. These data establish a strong CD8^+^ T cell component to the antitumor effects of depleting UNG in MC38 cells

### Effects of UNG knockdown in BALB/c mice inoculated with CT-26 colorectal tumors.

**Figure 8:**
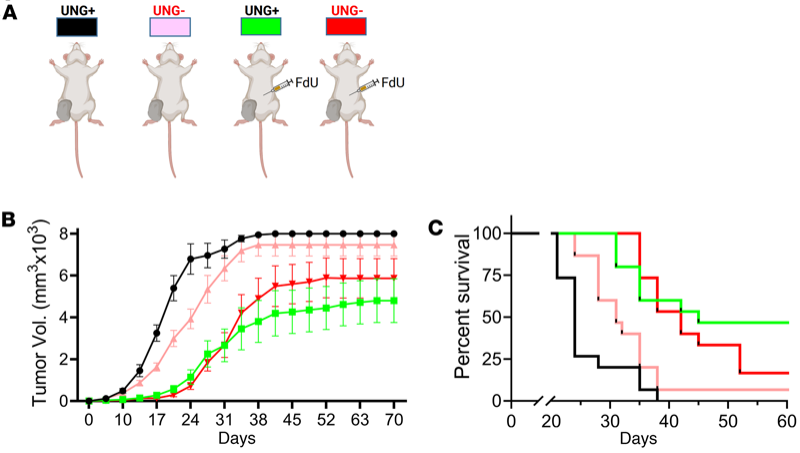
shRNA^UNG^ knockdown in BALB/c mice inoculated with CT-26 tumors. The antitumor effects of UNG protein shRNA depletion in the presence and absence of FdU treatment was investigated in the BALB/c MMR-competent mouse strain. CT-26 cells (5 × 10^5^) were injected into the right hind limb of female BALB/c mice. The experiment was performed twice, with 5 mice and 10 mice per group. (**A**) The 4 groups in each experiment were (a) shRNA^ctrl^ without FdU treatment, (b) shRNA^ctrl^ with FdU treatment, (c) shRNA^UNG^ without FdU treatment, and (d) shRNA^UNG^ with FdU treatment. The FdU treatment was performed in an identical fashion as described in the legend to Figure 3. (**B**) Tumor volumes for the 4 experimental groups were measured on the indicated days. Tumor volumes from the replicate experiments were combined and plotted for statistical analysis (*n* = 15 for each group). Error bars are SEM, and differences in tumor growth were compared using an unpaired 2-tailed *t* test. A significant reduction in tumor growth was observed for shRNA^ctrl^ + FdU vs. shRNA^ctrl^ (*P* = 0.0005), but depletion of UNG had no statistically significant benefit in this syngeneic model. (**C**) Kaplan-Meier survival analysis for the 4 experimental groups. Survival comparisons between groups used the log-rank Mantel-Cox test, which showed improved survival with shRNA^ctrl^ + FdU versus shRNA^ctrl^ (*P* = 0.0002). Depletion of UNG had no statistically significant benefit on survival in this syngeneic model.

Since different mouse syngeneic models can show different responses to chemotherapy and ICB, we explored the antitumor effects of UNG protein shRNA depletion in the presence and absence of FdU treatment in the MMR-competent CT-26 tumor syngeneic model grown in the BALB/c mouse strain, using the same doxycycline-inducible shRNA constructs, shRNA^UNG^ sequence A and shRNA^ctrl^. (We elected not to pursue the UNG inhibition approach with UGI in this mouse line because of the more favorable effects of UNG depletion observed in the MC38 line.) The experiment was performed twice, with 5 mice and 10 mice per group, respectively, and the tumor volumes and survival data from the replicate experiments were combined and plotted for statistical analysis (*n* = 15 for each group). The same test groups and FdU delivery protocols were used as described in the legend to Figure 5 for the MC38 mouse model (Figure 8A).

The experiment involved subcutaneous injection of CT-26 cells into the right hind limb of female BALB/c mice. In the absence of FdU, the shRNA^UNG^ test group showed modestly slower tumor growth and increased survival as compared with shRNA^ctrl^ (*P* = 0.022) (Figure 8, B and C). However, in the presence of FdU there was no statistically significant benefit of UNG depletion, although a significant reduction in tumor growth (*P* < 0.0001 and *P* < 0.0001) and increased survival (*P* < 0.0001 and *P* < 0.0001) were observed for both the shRNA^ctrl^ plus FdU and shRNA^UNG^ plus FdU groups as compared with shRNA^ctrl^. Thus, unlike the MC38 mouse model, depletion of UNG had minimal benefit in the CT-26 MMR-competent mouse model.

### Combining aPD1 therapy with UNG depletion delays CT-26 tumor growth in BALB/c mice.

**Figure 9:**
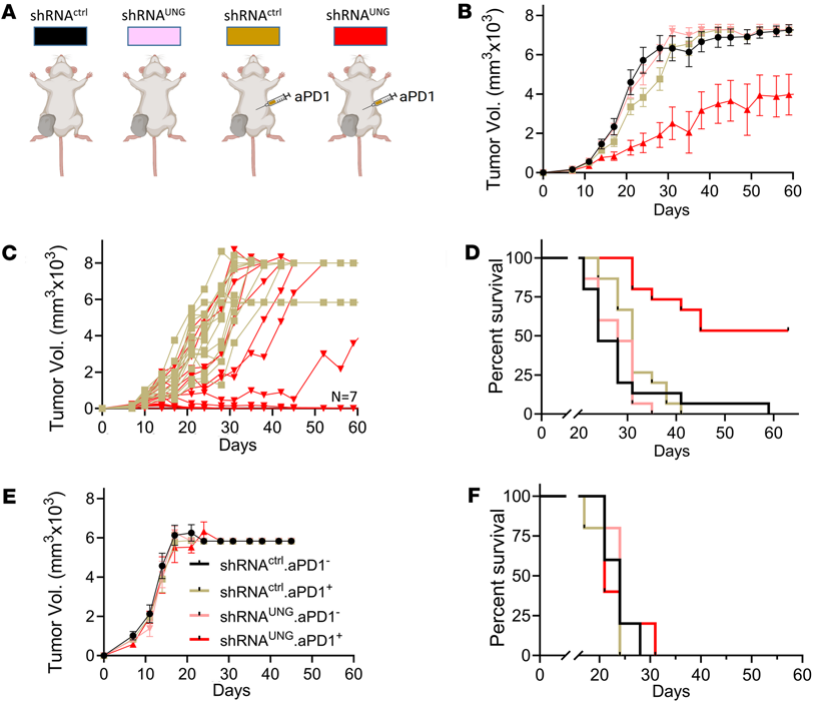
Anti–PD-1 therapy in the context of UNG depletion delays CT-26 tumor growth and increases BALB/c mouse survival in a CD8^+^ T cell–dependent manner. (**A**) BALB/c mice were treated with anti–PD-1 (aPD1) antibodies (0.1 mg twice weekly via intraperitoneal injection), with or without shRNA-mediated UNG depletion (shRNA^UNG^). Female mice were injected with 5 × 10^5^ CT-26 cells expressing either shRNA^UNG^ or shRNA^ctrl^, and the experiment was performed twice with 4 groups consisting of 5 mice each: (a) shRNA^ctrl^ without aPD1 antibodies (shRNA^ctrl^/aPD1^–^), (b) shRNA^ctrl^ + aPD1 antibodies (shRNA^ctrl^/aPD1^+^), (c) shRNA^UNG^ without aPD1 antibodies (shRNA^UNG^/aPD1^–^), and (d) shRNA^UNG^ + aPD1 antibodies (shRNA^UNG^/aPD1^+^). (**B**) Tumor growth for the 4 experimental groups was measured on the indicated days (colors correspond to **A**). Error bars for tumor growth are SEM, and the significance between groups was evaluated using an unpaired 2-tailed *t* test. A reduced growth rate for shRNA^UNG^/aPD1^+^ vs. shRNA^ctrl^/aPD1^+^ was observed (*P* = 0.026), while tumor growth in the mice receiving the 2 monotherapy regimes did not differ from the no-treatment control. (**C**) Tumor growth kinetics for individual CD8^proficient^ mice receiving the shRNA^ctrl^/aPD1^+^ (tan square) and shRNA^UNG^/aPD1^+^ (red triangle) treatments. (**D**) Survival of the 4 experimental treatment groups (colors correspond to **A**). Significance was tested using the log-rank Mantel-Cox test, which showed a significantly improved survival in CD8^proficient^ mice treated with shRNA^UNG^/aPD1^+^ vs. shRNA^ctrl^/aPD1^+^ (*P* = 0.005). (**E**) Tumor growth kinetics for CD8^depleted^ mice using the 4 treatments: shRNA^ctrl^/aPD1^–^ (black circle), shRNA^ctrl^/aPD1^+^ (tan square)**,** shRNA^UNG^/aPD1^–^ (pink triangle), and shRNA^UNG^/aPD1^+^ (red triangle). Tumors volumes were measured on the indicated days and the error bars are SEM. No significant differences in the tumor growth curves for the 4 groups of CD8^depleted^ mice were observed as determined by an unpaired 2-tailed *t* test (*P* > 0.05). (**F**) Survival of CD8^depleted^ mice using the 4 treatment modalities [shRNA^ctrl^/aPD1^–^ (black circle), shRNA^ctrl^/aPD1^+^ (tan square), shRNA^UNG^/aPD1^–^ (pink triangle), and shRNA^UNG^/aPD1^+^ (red triangle)] showed no significant differences as determined by an unpaired 2-sided *t* test (*P* > 0.05).

Given that UNG depletion did not result in a beneficial response in the CT-26 mouse model (Figure 8), and the previously reported immunogenicity of CT-26 tumors (25), we combined aPD1 with UNG depletion to explore whether PD-1–mediated T cell suppression might play a role in the poor response to UNG depletion alone. In the combination experiment, female BALB/c mice were injected with aPD1 antibodies twice weekly via intraperitoneal injection and 5 × 10^5^ CT-26 cells containing either shRNA^UNG^ or shRNA^ctrl^ were introduced into the right hind limb by subcutaneous injection. The experiment was performed twice with 4 experimental groups of 5 mice each (Figure 9A): (a) shRNA^ctrl^ without aPD1 antibodies (shRNA^ctrl^/aPD1^–^), (b) shRNA^ctrl^ with aPD1 antibodies (shRNA^ctrl^/aPD1^+^), (c) shRNA^UNG^ without aPD1 antibodies (shRNA^UNG^/aPD1^–^), and (d) shRNA^UNG^ with aPD1 antibodies (shRNA^UNG^/aPD1^+^). Tumor growth measurements taken over 60 days showed no significant differences between the no-treatment control and the 2 monotherapy groups (shRNA^ctrl^/aPD1^+^ and shRNA^UNG^/aPD1^–^) (Figure 9, B and C). In contrast, the shRNA^UNG^ plus aPD1 combination therapy group showed reduced tumor growth as compared with the 2 monotherapy groups (shRNA^ctrl^/aPD1, *P* = 0.06; and shRNA^UNG^/aPD1^–^, *P* = 0.024). A survival analysis of the 4 experimental groups showed significantly improved survival with the combination regimen as compared with the no-treatment control and aPD1 monotherapy alone (*P* = 0.005) (Figure 9D).

### CD8^+^ depletion ablates the benefit of aPD1 therapy with UNG depletion in CT-26 tumors.

To determine whether the synergy observed between aPD1 therapy and UNG depletion in the CT-26 tumor model was CD8^+^ T cell dependent, the depletion of cytotoxic CD8^+^ T cells was performed in combination with aPD1 and/or UNG depletion. Accordingly, the test groups were (a) shRNA^ctrl^ without CD8^+^ cell depletion (shRNA^ctrl^ CD8^proficient^), (b) shRNA^ctrl^ with CD8^+^ cell depletion (shRNA^ctrl^ CD8^depleted^), (c) shRNA^ctrl^/aPD1^+^ without CD8^+^ cell depletion (shRNA^ctrl^/aPD1^+^ CD8^proficient^), (d) shRNA^ctrl^/aPD1^+^ with CD8^+^ cell depletion (shRNA^ctrl^/aPD1^+^ CD8^depleted^), (e) shRNA^UNG^ without CD8^+^ cell depletion (shRNA^UNG^ CD8^proficient^), (f) shRNA^UNG^ with CD8^+^ cell depletion (shRNA^UNG^ CD8^depleted^), (g) shRNA^UNG^/aPD1^+^ without CD8^+^ cell depletion (shRNA^UNG^/aPD1^+^ CD8^proficient^), and (h) shRNA^UNG^/aPD1^+^ with CD8^+^ cell depletion (shRNA^UNG^/aPD1^+^ CD8^depleted^). Consistent with a key role of CD8^+^ T cells in the observed antitumor effects of combined aPD1 and UNG knockdown, there was no difference in tumor volumes between shRNA^ctrl^ alone and those with aPD1 treatment and/or UNG depletion (Figure 9, E and F). As was observed in prior experiments in the CD8^proficient^ background, there was significantly reduced tumor growth and improved murine survival when mice with CT-26 tumors had combined shRNA^UNG^ and aPD1. These data establish a strong CD8^+^ T cell component to the antitumor effects of aPD1 and UNG depletion in the CT-26 tumor model.

## Discussion

The key findings in this study provide the first evidence to our knowledge that inhibition or depletion of UNG in the presence and/or absence of FdU can provide a beneficial therapeutic effect through increased tumor immunogenicity in mice. This result was particularly unexpected because the mouse MC38 and CT-26 CRC tumor lines do not show any sensitivity to UNG inhibition or depletion in the context of in vitro experiments (Supplemental Figures 1 and 2). Thus, the antitumor phenotypes resulting from UNG inhibition or protein depletion arise from changes that promote immune activation in mice, rather than induced tumor cell toxicity. This conclusion is supported by analyses of the changes in immune cell populations induced by UNG inhibition/depletion, in the presence and absence of FdU, and the requirement for a CD8^+^ T cell response (Figure 7). We had previously proposed, based on the increased FdU sensitivity of many human CRC lines that when UNG was inhibited (3), a therapeutic effect would be manifested only in cell lines that showed increased in vitro sensitivity to UNG inhibition. Thus, the unanticipated behavior of these 2 mouse cell lines suggests that in vivo UNG-related human cancer cell killing could involve both an intrinsic cellular component (such as the cellular DDR phenotype) and an immune component that is only manifested in vivo.

### Inhibition and depletion of UNG are not equivalent in the two mouse syngeneic models.

A key result in this study was that the MC38 MMR-deficient cell line showed a modest FdU-dependent sensitivity to UNG inhibition (Figure 3), while shRNA depletion of UNG was sufficient to bring about FdU-independent tumor clearance when MC38 cells were introduced into C57BL/6J mice (Figure 5). In contrast, depletion of UNG in MMR-competent CT-26 cells had no statistically significant benefit on tumor growth or survival in the BALB/c mouse strain (Figure 8). However, when combined with PD-1 checkpoint blockade, UNG depletion in CT-26 tumors increased survival as compared with the no-treatment control and aPD1 monotherapy alone, supporting the conclusion that immune activation can contribute a therapeutic effect in both syngeneic mouse models under appropriate conditions (Figure 9). Understanding the mechanistic bases for these responses will require further studies beyond the scope of the current work.

We speculate that addition of FdU is required for the modest therapeutic effect of UNG inhibition in the MC38-C57BL/6J mouse model because an increased burden of DNA damage lesions is needed to induce the replication stress when UNG activity is inhibited. In contrast, depletion of the protein, which includes removal of its PCNA and RPA interaction domains, may introduce marked replication stress even in the absence of FdU. This type of mechanism is consistent with the known contributions of UNG to replication fork stability (4, 13). Depletion of UNG in CT-26 cells had only a small effect on tumor growth and survival in BALB/c mice, suggesting that the UNG-associated replication stress threshold in this cell line is higher than DDR-deficient MC38 cells. Indeed, in the CT-26–BALB/c model, tumor growth and survival was dominated by the effects of FdU alone (e.g. in the absence of PD-1 ICB).

### Possible immunogenicity mechanisms.

The tumor immunogenicity associated with UNG inhibition or depletion appears on the time scale of several days as judged by tumor growth measurements (Figures 3 and 5). Given this fairly rapid onset, and the knowledge that replication fork arrest occurs with FdU incorporation into DNA or deletion of the N-terminal domain of UNG (4, 6, 13), we favor an immune activation mechanism involving release of immunogenic ssDNA from stalled forks. Although we did not observe an upregulated interferon response using RT-qPCR upon in vitro depletion of UNG in MC38 and CT-26 cells (Supplemental Figure 4), it is possible that the immune activation mechanism requires replication stress in the context of the in vivo syngeneic mouse model. Immune activation is consistent with our CyTOF analyses, where M1 macrophages, DCs, and Th cells were elevated in the context of combined UNG inhibition and FdU therapy (Figure 4). In addition, decreases in antiinflammatory M2 macrophages were generally observed (Figures 4 and 6). Although an alternative mechanism involving an increase in the mutational burden and resulting generation of tumor-associated neoantigens cannot be rigorously excluded based on the current findings, a replication stress mechanism seems more likely.

By comparing the cytotoxic effects of dU, BrdU, and FdU in the presence and absence of UNG activity in human cancer cells, we have previously proposed that the incorporation and persistence of FdU in DNA is uniquely cytotoxic. Remarkably, treating human cells with millimolar concentrations of dU (which is converted into dUTP) proved nontoxic regardless of hUNG activity, indicating that the presence of genomic U/A pairs, or the excision of uracil, is not toxic (3). This result leads to the broader conclusion that increased dUTP levels and U/A pairs caused thymidylate synthase (TS) inhibitors such as pemetrexed or raltitrexed are not intrinsically toxic. Thus, the toxicity mechanism of these antifolate drugs must derive from depletion of the dTTP pool through TS inhibition, rather than uracil excision by UNG. Further support for the proposal that halopyrimidine base excision is not required for cytotoxicity is provided by the TS inhibitor BrdU, which shows cellular toxicity at concentrations 100-fold lower than dU, but is not a substrate of UNG (3). We hypothesize that the unique chemical properties of FdU, which result in both potent TS inhibition and high levels of FU/G pairs in DNA (due to the mispairing properties of the FU base) (26, 27), make FdU especially toxic to replication forks and immunogenic in the absence of UNG excision.

### Implications for human cancer therapy.

There has been great interest as well as encouraging results using aPD1 therapy for the treatment of microsatellite instability–high (MSI-H) CRC (28). Several papers have now reported high rates of complete pathological response with ICB in early-stage MSI-H CRCs (19, 29). Although only a small fraction of CRCs are MSI-H, these results established the potential of targeting faulty DNA repair phenotypes in cancer. Extending the use of ICB to other cancers by combining with chemotherapy or targeted therapies is an area of active research. A recent phase III clinical trial combined nivolumab and FU/cisplatin in the treatment of metastatic oesophageal squamous cell carcinoma and observed enhanced progression-free and overall survival for the combination regimen (30, 31). The favorable results reported here by combining UNG depletion with aPD1 antibodies suggests that UNG inhibition/depletion may have synergistic effects with ICB strategies for microsatellite stable (MSS) disease.

Contrasting with the success of immunotherapy, several studies have indicated a reduced or absent clinical benefit of FU monotherapy in the context of MSI-H CRC. MSS disease displays the opposite relationship, with increased FU responsiveness and almost universal resistance to aPD1 monotherapy (32, 33). These different therapeutic vulnerabilities may relate to different DDR capacities in these CRC subtypes that could be exploited with the targeting methods suggested above. The MSI-H phenotype has a higher propensity for persistent incorporation of mutagenic bases (such as FU/G pairs), while MSS tumors have the DNA repair machinery to remove these lesions. Since FU monotherapy leads to only modest levels of FU/G pairs in DNA, these may be easily tolerated in repair-deficient MSI-H CRC, while their excision via the MMR pathway may provoke strand breaks and replication fork arrest in MSS disease (33). These plausible mechanisms could account for the observed clinical sensitivities of MSI-H and MSS cancers to FU.

Unlike FU, the FdU nucleoside is associated with much higher levels of FU deoxynucleotide incorporation into DNA, which would lead to the persistence of high levels of immunogenic FU/G mismatches in MSI-H tumors, especially in the context of UNG inhibition/depletion (3). Interestingly our data demonstrate enhanced immunogenicity with depletion of UNG using shRNA in the absence of any FP drug, particularly in the MSI-H MC38 background (15). UNG has an important role in replication fork stability independent of its catalytic activity, which may explain why depletion alone is sufficient to promote tumor clearance in the MC38 mouse model and is synergistic with aPD1 in CT-26 cells (6). The different effects observed here with UNG inhibition as compared with depletion also suggests that small-molecule inhibitors and/or UNG protein degraders may have distinct and differential clinical benefits and uses (35). These proposed mechanisms will be the focus of ongoing studies in this area.

## Methods

### Sex as a biological variable.

The syngeneic murine CRC models (MC38 and CT-26) utilized in this study are both derived from female mice and therefore all murine experiments were performed in female mice. This was done as these are the best characterized syngeneic murine CRC models available. While we do not anticipate murine (or patient sex) will influence the results observed in these experiments we acknowledge that this was not a variable that we were able to effective assess in these model systems.

### Chemicals.

FdU was obtained from Tocris. Puromycin and blastocidin were obtained from Sigma-Aldrich.

### Antibodies.

aPD1 (clone RMP1-14, catalog BE0146), anti-CD8 (clone 2.43, catalog BE0061), IgG2a isotype control (clone 2A3, catalog BE0089), and IgG2b isotype control (clone LTF-2, catalog BE0090) antibodies were obtained from Bio X Cell.

### Animals.

Wild-type C57BL/6J and BALB/c mice were purchased from The Jackson Laboratory. For syngeneic mouse models, age-matched 8- to 12-week-old female mice were injected with MC38 (2.5 × 10^5^ cells) or CT-26 (5 × 10^5^ cells) subcutaneously into the right hind limb of C57BL/6J or BALB/c mice, respectively. Given the lack of syngeneic CRC models in male mice, we only used female mice for these experiments. We do not expect the effects of UNG inhibition/depletion to differ by sex and anticipate these results will be generalizable to male subjects. For tumor growth and survival experiments, tumors were measured twice weekly with sacrifice of mice when tumors reached 20 mm in greatest diameter or impeded lower extremity function. The details of each murine experiment are discussed in dedicated sections of the methods.

### Mouse cell lines.

MC38 and CT-26 syngeneic murine cell lines were established and maintained in the laboratory of Elizabeth Jaffee (Johns Hopkins University).

### DNA oligonucleotides and DNA sequencing.

All DNA oligonucleotide primers and substrates were synthesized by Integrated DNA Technologies (IDT). Sanger DNA sequencing was performed by the Johns Hopkins University sequencing core. Primer and shRNA guide sequences are listed in Supplemental Table 1.

### FdU concentration-response studies.

MC38 and CT-26 cells were grown to 50% confluence in T25 flasks prior to addition of 0.05% trypsin to detach the adherent cells. Cells were then split into 2 T25 flasks, one of which contained media with tetracycline-free FBS, and the other contained media with 0.5 mg/mL doxycycline. After 48 hours of growth, cells were detached using trypsin and passed through a 35-μm filter (ThermoFisher) to eliminate cell clumps prior to counting with a hemacytometer. The 96-well plates were then seeded at a density of 2000 cells per well, with the top half of each plate containing the cells grown in tetracycline-free conditions (UNG-proficient) and the bottom half containing cells grown with doxycycline-containing media (UNG-inhibited). After 24 hours, cell cultures (in triplicate) were exposed to phenol red–free growth media containing 90 fM to 100 mM FdU in log 3 increments. For the induced cell group, doxycycline was included in the media at 0.5 mg/mL for the entire period of drug treatment. Cells were incubated in a humidified incubator for a total of 72 hours. After this period, 10 mL of CellTiter 96 Aqueous MTS reagent and phenazine ethosulfate solution (Promega) were added to each well. Absorption measurements were made at 492 nm using a Model M200 Pro plate reader (Tecan) at 30, 60, and 120 minutes after addition of the MTS assay reagents. The concentration-response data in the absence and presence of UNG activity were fitted to Eq. 1 using GraphPad Prism software, where C is an offset, A is the total amplitude for the loss in cell viability, EC_50_ is the drug concentration at which one-half of the total viability loss occurs, [X] is the drug concentration, and *n* is the Hill slope parameter:

Percentage viable cells = C + [A/(1 +10^[log(EC50^^–^^[X])^^×^^n]^)] × 100 Eq. 1

### Construction of doxycycline-inducible UGI cell lines by lentiviral transduction.

The cDNA sequence of the UGI derived from bacterial phage PBS2 was synthesized by IDT (3). This sequence was inserted into the pENTR4 plasmid from Addgene and transferred into the CW57.1 doxycycline-inducible overexpression plasmid (Addgene) using the Gateway recombinase system from Thermo Fisher Scientific. Sanger sequencing was performed at the conclusion of each step to confirm the expected product. The CW57.1 UGI plasmid was then transfected into T293-FT cells along with pspax2 and pmd2.g helper vectors (Addgene). Over the 96 hours after transfection, the virus-containing supernatant was harvested and concentrated 100-fold. The viral supernatant was used to transduce the MC38 and CT-26 mouse cell line. After allowing 72 hours for plasmid incorporation, cells were selected with 1 μg/mL puromycin for 1 week. To confirm successful incorporation, cellular lysates were prepared from these cells grown either in tetracycline-free media or media containing 0.5 μg/mL doxycycline to induce UGI expression. UNG activity was then determined under these conditions using our UNG activity assay (see below).

### Targeted shRNA depletion of UNG in mice.

Sequences for shRNA guides targeting *UNG* as well as a nontargeting control were synthesized by IDT and inserted into the EZ-Tet-pLKO-Blast plasmid from Addgene. Sanger sequencing confirmed the correct products. The pLKO.1 G418 plasmid was then transfected into T293-FT cells along with helper plasmids pspax2 and pmd2.g. Virus was harvested and titered as described above. These lentiviral particles were used to infect the MC38 and CT-26 cell lines. After blasticidin (10 μg/mL) selection for 2 weeks, cellular lysates were prepared from these cells grown in either tetracycline-free media or media containing 0.5 μg/mL doxycycline to induce shRNA expression. Efficient UNG knockdown was confirmed by activity measurements using cell lysates from cells grown under these conditions (see below).

### UNG activity in cell extracts.

All cell lines containing the CW57.1 UGI construct were plated at a density of 1 × 10^5^ cells per T25 flask and grown to 80% confluence in either tetracycline-free media (A4736401, Gibco) or media containing 0.5 mg/mL doxycycline (Tocris). Unmodified parental cells were also grown to 80% confluence in tetracycline-free media. Cell extracts were obtained under native conditions by dissociating cells from the flask surface using 0.05% trypsin. Cells were then pelleted and washed 3 times with PBS. Cell pellets were then resuspended in 100 mL CelLytic MT (Sigma-Aldrich). Cells were then incubated at 4°C for 30 minutes to allow for cell lysis. This solution was then centrifuged at 21,000*g* for 20 minutes. In total, 75 mL of supernatant was then removed and centrifuged at 21,000*g* for 5 minutes, and 50 mL was removed for use in downstream assays and protein concentrations determined using the Bradford colorimetric protein assay with BSA as the standard (Thermo Fisher Scientific).

### Survival experiments for UNG inhibition and/or UNG depletion in combination with FdU.

Age-matched 8- to 12-week old female mice were injected with MC38 (2.5 × 10^5^ cells) or CT-26 (5 × 10^5^ cells) subcutaneously into the right hind limb of C57BL/6J or BALB/c mice, respectively. Mice were divided into treatment groups and where UNG perturbation or control shRNA induction was desired, mice switched on day 0 to drinking water containing 2 mg/mL doxycycline to drink to thirst. Starting on day 3, mice in FdU treatment groups were treated with FdU injected intraperitoneally daily at 50 mg/kg for C57BL/6J or 25 mg/kg for BALB/c mice through day 12. Mice not receiving FdU underwent intraperitoneal PBS injections on the same schedule. Tumors were measured twice weekly. For the MC38 model, we did a repeat experiment comparing mice with tumors containing a different shRNA guide targeting UNG (sequence B) with tumors containing the control shRNA guide. All subsequent experiments were done with shRNA^UNG^ (sequence A). Mice were sacrificed when tumors reached 20 mm in the largest dimension or impeded limb movement.

### Survival experiments for CD8^+^ lymphocyte depletion and UNG depletion in MC38 cells.

Age-matched 8- to 12-week-old female C57BL/6J mice were divided in half and given 2 intraperitoneal doses in the week before tumor inoculation of either 200 μg of anti-CD8^+^ antibodies, or isotype rat IgG2b control antibody creating 2 groups (CD8^depleted^ and CD8^proficient^) of 10 mice. Following this week, half of the mice in each group were injected with MC38 (2.5 × 10^5^ cells) modified to either express the UNG shRNA construct (sequence A) or an shRNA nontargeting control construct subcutaneously into the right hind limb. This created the 4 following treatment groups: (a) shRNA^ctrl^ CD8^depleted^, (b) shRNA^UNG^ CD8^depleted^, (c) shRNA^ctrl^ CD8^proficient^, and (d) shRNA^UNG^ CD8^proficient^. Mice were switched on day 0 to drinking water containing 2 mg/mL of doxycycline to drink to thirst. CD8^+^ depletion or control antibodies were continued with biweekly 100-μg doses for 4 weeks after inoculation as per original group assignment. Tumors were measured twice weekly. Mice were sacrificed when tumors reached 20 mm in the largest dimension or impeded limb movement.

### Survival experiments for aPD1 treatment and UNG depletion in CT-26 cells.

Age-matched 8- to 12-week-old female BALB/c mice were injected with CT-26 (5 × 10^5^ cells) modified to either express the UNG shRNA construct (sequence A) or an shRNA nontargeting control construct subcutaneously into the right hind limb. Mice were divided into the following treatment groups: (a) shRNA^ctrl^/aPD1^–^, (b) shRNA^UNG^/aPD1^–^, (c) shRNA^ctrl^/aPD1^+^, and (d) shRNA^UNG^/aPD1^+^. Mice were switched on day 0 to drinking water containing 2 mg/mL of doxycycline to drink to thirst. Mice were treated with 100 μg aPD1 or IgG2 isotype control antibodies biweekly for 4 weeks after inoculation. Tumors were measured twice weekly. Mice were sacrificed when tumors reached 20 mm in the largest dimension or impeded limb movement.

### Survival experiments for CD8^+^ lymphocyte depletion and aPD1 and UNG depletion in the CT-26 tumor model.

Age-matched 8- to 12-week-old female BALB/c mice were divided in half and given 2 intraperitoneal doses in the week before tumor inoculation of either 200 μg of anti-CD8 or isotype rat IgG2b control antibody creating 2 groups (CD8^depleted^ and CD8^proficient^) of 20 mice. Following this week, half of the mice in each group were injected with CT-26 (5 × 10^5^ cells) modified to either express the UNG shRNA construct (sequence A) or an shRNA nontargeting control construct subcutaneously into the right hind limb. Each group was then further divided in half into groups treated with aPD1 and those treated with isotype rat IgG2α control antibody. This created the following 8 treatment groups: (a) shRNA^ctrl^ CD8^depleted^, (b) shRNA^UNG^ CD8^depleted^, (c) shRNA^ctrl^/aPD1^+^ CD8^depleted^, (d) shRNA^UNG^/aPD1^+^ CD8^depleted^, (e) shRNA^ctrl^ CD8^proficient^, (f) shRNA^UNG^ CD8^proficient^, (g) shRNA^ctrl^/aPD1^+^ CD8^proficient^, and (h) shRNA^UNG^/aPD1^+^ CD8^proficient^. Mice were switched on day 0 to drinking water containing 2 mg/mL doxycycline to drink to thirst. CD8^+^ cell depletion or control antibodies were continued with biweekly 100 μg doses for 4 weeks after inoculation as per original group assignment. aPD1 or control antibodies were continued with biweekly 100 μg doses for 4 weeks after inoculation as per original group assignment. Tumors were measured twice weekly. Mice were sacrificed when tumors reached 18 mm in the largest dimension or impeded limb movement.

### CyTOF analysis of immune cell populations.

Age-matched 8- to 12-week-old female C57BL/6J mice were used for these experiments. For UGI tumor microenvironment experiments, mice were injected with MC38 (2.5 × 10^5^ cells) modified to contain the CW57.1 UGI construct subcutaneously into the right hind limb. Mice were divided into the following 4 treatment groups: (a) intact UNG, (b) inhibited UNG, (c) intact UNG/FdU treatment, (d) inhibited UNG/FdU treatment. In the arms with UNG inhibition, mice switched on day 0 to drinking water containing 2 mg/mL doxycycline to drink to thirst. Mice were treated with daily intraperitoneal FdU (50 mg/kg) or PBS injections on days 3 to 12. Tumors were allowed to grow until day 20 before mice were sacrificed and tumors excised. Tumors were profiled as previously described in detail (36). Briefly, tumors were minced for enzymatic dissociation into single-cell suspensions using a gentleMACS Tumor Dissociation Kit (Miltenyi Biotec). Resulting samples were filtered and enriched using a Percoll gradient (40% over 80%). Cells from each tumor dissociation were washed with Cell Staining Buffer (CSB, Standard Biotools) stained for viability using 500 nM palladium (Sigma-Aldrich) in Maxpar PBS (Standard Biotools), barcoded for 10-plex multiplexing, after which the batches were stained together with Fc blockade for 10 minutes at room temperature followed by a cocktail of surface staining antibodies (Supplemental Table 2) for 30 minutes at room temperature. After Fix/Perm (eBiosciences), following the manufacturer’s protocol, batches were stained with a cocktail of intracellular antibodies for 30 minutes at room temperature. Completely stained samples were then fixed with freshly prepared 1.6% paraformaldehyde (Thermo Fisher Scientific) in Maxpar PBS and stored overnight at 4°C. All cells were labeled with 1:500 rhodium in Maxpar Fix & Perm Buffer (Standard Biotools), rinsed with CSB, and washed with Maxpar Water (Standard Biotools). All data were acquired using a Helios Mass Cytometer (Standard Biotools) at the Johns Hopkins Mass Cytometry Facility. Upon preprocessing (data normalization and randomization) with CyTOF software (v7.0.5189) (Standard Biotools), cell events were gated based on size and viability and then de-barcoded using FlowJo (v10.6) (BD Biosciences).

For the shRNA tumor microenvironment experiments, mice were injected with either MC38 (2.5 × 10^5^ cells) modified to either express the shRNA^UNG^ construct (sequence A) or an shRNA^ctrl^ construct subcutaneously into the right hind limb. Mice were switched on day 0 to drinking water containing 2 mg/mL doxycycline to drink to thirst. Tumors were allowed to grow for 1 week before mice were sacrificed and tumors excised. Given the visualized size difference in excised tumors between the shRNA^UNG^ group and shRNA^ctrl^ group, the excised tumors from each group were weighed (Supplemental Figure 5).

### IMC staining and image acquisition.

Slides were baked for 2 hours in preheated oven at 60°C, deparaffinized with xylene for 20 minutes with light agitation, and rehydrated using an alcohol gradient (100%, 95%, 80%, and 70% ethanol for 5 minutes each). The slides were washed in Maxpar water (Standard Biotools) and then placed in target retrieval solution (Antigen Retrieval Agent, pH 9, 1:10 in Maxpar water) equilibrated at sub-boiling temperature (90°C–95°C) for 1 hour followed by Maxpar PBS wash for 10 minutes with gentle agitation. Sections were blocked using 3% BSA for 45 minutes at room temperature followed by incubation in the IMC antibody cocktail solution (Supplemental Table 3) overnight at 4°C. For nuclear counterstaining, sections were stained with Cell-ID Intercalator-Ir (Standard Biotools) diluted at 1:400 in Maxpar PBS for 30 minutes at room temperature. Tissues were counterstained with 0.5% ruthenium tetroxide (Electron Microscopy Sciences, 20700-05) at 1:2000 for 3 minutes at room temperature. After washing in Maxpar water, slides were air-dried and loaded in the Hyperion Imaging Plus System (Standard Biotools) for image acquisition at the Johns Hopkins Mass Cytometry Facility. Regions of interest with representative tumor tissue were selected for ablation based on H&E.

### IMC data preprocessing and analysis.

Representative images were created and exported using MCD Viewer 1.0560.2 (Standard Biotools). A software pipeline based on DeepCell (https://deepcell.org/) and HistoCAT 1.76 (https://bodenmillergroup.github.io/histocat-web/) was used for image segmentation. Single-cell datasets were imported into R 4.2.2 for further analysis (37, 38). Clusters were generated with PhenoGraph and annotated based on expression profiles of canonical markers (Supplemental Figure 6) (39). The *x* and *y* coordinates of each cell were used for calculating distance relationships using spatstat (http://spatstat.org/). Plots were generated using ggplot (https://ggplot2.tidyverse.org/) and igraph (https://igraph.org/).

### RT-qPCR measurements of IFN response gene expression.

For this investigation, we looked at a panel of IFN-associated genes (STING, IFN-α, STAT1, IFN-β, IL-6, MDA5, cGAS, ISG15, RANTES, and MX2) to compare pre- and post-shRNA induction levels for the shRNA control and UNG shRNA (sequence A) cell lines in the MC38 and CT-26 background. This comparison was chosen in order to validate whether an IFN-stimulated immune response was triggered by shRNA knockdown of UNG compared to controls.

### RNA isolation.

Cells were grown to 80% confluence in T-75 flasks and then 1 μg/mL doxycycline was added to one flask to induce shRNA^UNG^ or shRNA^ctrl^ production while the other flask was left in base media for uninduced controls. Cells were incubated for 24 hours and then released from the flask using trypsin. A QIAwave RNA Mini kit (Qiagen) was used to extract RNA from cells and total RNA yield was quantified by A260 absorbance using a NanoDrop 2000 (Thermo Fisher Scientific).

### Reverse transcription and RT-qPCR.

For reverse transcription, a high-capacity cDNA kit (Invitrogen) was used with an input of 22 μg of total cellular RNA. The reverse transcription reactions were thermocycled for 10 minutes at 25°C, 120 minutes at 37°C, and finally 5 minutes at 85°C. qPCR was performed in technical triplicate for 3 independently prepared total RNA isolates. Reactions contained 5 μL of a 500 nM mix of forward and reverse primers, 5 μL of cDNA input, and 10 μL of 2× QuantiTect SYBR Green Master Mix (Qiagen). The reactions were then thermocycled in a RotorGeneQ (Qiagen) with an initial 15-minute activation at 95°C followed by 45 cycles (94°C for 15 seconds, 55°C for 30 seconds, 72°C for 30 seconds). Raw fluorescence was read after each cycle to determine the Ct values. Ct values for each interferon gene of interest (GOI) (Supplemental Table 4) were normalized to the GAPDH control gene by calculating ΔCt using the following equation: ΔCt = Ct(GOI) − Ct(GAPDH). The ΔCt values for the induced shRNA^UNG^ or shRNA^CTRL^ and noninduced cell lines were compared using the ΔΔCt method with the following equation: ΔΔCt = ΔCt(induced)−ΔCt (noninduced). The ΔΔCt values were converted to fold changes using the following equation: fold change = 2^−ΔΔCt^.

### Statistics.

Data were analyzed using the Prism 9.0 statistical program from GraphPad Software. The uncertainties in the IC_50_ values derived from the concentration-response curves are expressed as mean ± SD and are derived from at least 3 biologic replicates for cellular experiments, each performed with 3 technical replicates. Dose-response curves were generated using variable slope (4 parameter) calculations in Prism. Kaplan-Meier survival curves were created for each murine survival experiment with survival comparisons made using the log-rank Mantel-Cox test. CyTOF data were analyzed and visualized by R (v4.3.0). Preprocessed cells were clustered using FlowSOM and annotated into cell types based on expression profiles of canonical markers (40). Abundances were compared between 2 groups by 2-tailed *t* tests, and *P* values less than 0.05 were considered significant. When 4 treatment groups were present, *P* values were adjusted for multiple comparisons within each cell type using Benjamini-Hochberg.

### Study approval.

All murine experiments were performed in the Johns Hopkins University cancer research building 2 animal core facility (Baltimore, MD). Animal experiments were performed in accordance with the Johns Hopkins School of Medicine Animal Care and Use Committee–approved protocol MO19M170. Studies complied with all ethical regulations regarding animal research.

### Data availability.

Values for all data points in graphs are reported in the Supporting Data Values file. All underlying raw and processed data has been deposited to a server at Johns Hopkins and are available upon request.

## Author contributions

ESC, BES, and JTS designed the research studie. ESC, BES, TJN, SC, NEG, SMS, AH, and WJH conducted experiments. ESC, BES, TJN, SC, NEG, SMS, AH, and WJH acquired data. All authors analyzed data. VY and JTS provided reagents. All authors contributed to writing the manuscript.

## Supplementary Material

Supplemental data

Unedited blot and gel images

Supporting data values

## Version 1 07/15/2025

In-Press Preview

## Version 2 08/22/2025

Electronic publication
