# Supplementary material for: Ablating UNG activity in a mouse model inhibits colorectal cancer growth by increasing tumor immunogenicity
Source: JCI Insight. 2025 Jul 15;10(16):e184435. doi: 10.1172/jci.insight.184435 (PMC12406714; doi:10.1172/jci.insight.184435)
Supplement: Supplemental data [file jciinsight-10-184435-s099.pdf]

Supplemental Information

Ablation of UNG activity in a syngeneic mouse model inhibits colorectal cancer growth by increasing tumor immunogenicity

Eric S. Christenson[§,#*], Brandon Smith[†], Thanh Nguyen[§,#], Soren Charmsaz[§], Nicole E. Gross[§], Sarah M. Shin[§], Alexei Hernandez[§], Won Jin Ho[§,#], Vasan Yegnasubramanian[§,#] and James T. Stivers[†]

[†]Department of Pharmacology and Molecular Sciences, Johns Hopkins University School of Medicine 725 North Wolfe Street Baltimore, MD 21205

[§]Department of Oncology, Sidney Kimmel Comprehensive Cancer Center at Johns Hopkins, Johns Hopkins University School of Medicine, 1650 Orleans Street, CRB1 Baltimore, MD, 21287 USA

[#]Cancer Convergence Institute at Johns Hopkins, Johns Hopkins University School Medicine, Baltimore, MD 21287, USA

Supplemental Tables

**Supplemental Table S1.** Nucleotide sequences for shRNAs, UGI protein and primers for DNA sequencing		
Name	Purpose	Sequence
shRNActrl	Non-targeting shRNA	TAAGGCTATGAAGAGATAC
shRNAUNG [sequence A]	UNG shRNA knockdown	CGTCAAGCTAATGGGATTTGT
shRNAUNG [sequence B]	UNG shRNA knockdown	TGACACGGATAGATCTCTATA
shRNA seq primer for	Sequencing primer	GAGGGCCTATTTCCCATGATT
shRNA seq primer rev	Sequencing primer	GACTATCATATGCTTACCGT
UGI	Dox inducible inhibition of UNG(3)	ATGACTAACCTGTCCGATATCATCGAAAAAGAGACTGGC AAACAGCTGGTCATCCAGGAGTCCATCCTGATGCTGCCT GAAGAGGTGGAGGAGGTCATTGGCAACAAGCCCGAGA GCGATATCCTGGTCCACACCGCCTACGACGAGTCCACC GACGAGAATGTGATGCTGCTCACCTCTGACGCCCCCGA GTATAAACCATGGGCTCTCGTGATCCAGGACAGTAACG GGGAGAACAAGATCAAGATGCTGTGA
UGI	Sequencing primer(3)	TGAACCGTCAGATCGCCTGG

Supplemental Table 2. Antibodies Used for Mass Cytometry					
Surface Stain					
Channel	Antigen	Clone	Source	Custom?	Dilution
141	Ly6G	1A8	Standard BioTools™		1:50
142	CD11c	N418	Standard BioTools™		1:100
143	CD69	H1.2F3	Standard BioTools™		1:100
145	CD4	RM4-5	Standard BioTools™		1:200
146	F4/80	BM8	Standard BioTools™		1:100
147	CD45	30-F11	Standard BioTools™		1:200
148	CD11b	M1/70	Standard BioTools™		1:100
149	CD19	6D5	Standard BioTools™		1:100
150	Ly6C	HK1.4	Standard BioTools™		1:100
151	CD25	3C7	Standard BioTools™		1:100
152	CD3e	145-2C11	Standard BioTools™		1:50
153	PDL1	10F.9G2*	Standard BioTools™		1:100
154	CTLA4	UC10-4B9	Standard BioTools™		1:100
156	BTLA	6F7	Standard BioTools™		1:100
158	KLRG1	2F1	MilliporeSigma™	X	1:400
159	PD1	29F.1A12	Standard BioTools™		1:100
161	CD40	HM40-3	Standard BioTools™		1:50
163	CD86	GL-1	Biolegend®	X	1:400
164	CD62L	MEL-14	Standard BioTools™		1:100
168	CD8a	53-6.7	Standard BioTools™		1:50
170	NK1.1	PK136	Standard BioTools™		1:50
171	CD44	IM7	Standard BioTools™		1:100

174	Lag3	C9B7W	Standard BioTools™		1:100
175	CD80	16-10A1	Biolegend®	X	1:400
176	B220	RA3-6B2	Standard BioTools™		1:200
209	I-A/I-E	M5/114.15.2	Standard BioTools™		1:100
**Intracellular Stain**					
**Channel**	**Antigen**	**Clone**	**Source**	**Custom?**	**Dilution**
154	CTLA4	UC10-4B9	Standard BioTools™		1:200
155	IRF4	3E4	Standard BioTools™		1:100
160	Tbet	4B10	Standard BioTools™		1:100
162	Ki-67	B56	Standard BioTools™		1:100
165	Foxp3	FJK-16s	Standard BioTools™		1:33
166	RORγt	B2D	eBioscience™	X	1:100
167	EOMES	Dan11mag	eBioscience™	X	1:200
169	CD206	C068C2	Standard BioTools™		1:100
172	Perforin	OMAK-D	Standard BioTools™		1:100
173	Granzyme B	GB11	Standard BioTools™		1:66
**Barcoding**					
**Channel**	**Antigen**	**Clone**	**Source**	**Custom?**	**Dilution**
112-116	CD98	RL388	Biolegend®	X	1:200
112-116	CD45	30-F11	Biolegend®	X	1:200

Supplemental Table 3: Antibodies Used for IMC Staining					
**Channel**	**Antigen**	**Clone**	**Dilution**	**Vendor**	**Custom**
89	CD45	2B11 + PD7/26	250	CST	X
96-104	Ruthenium-Tissue Counterstain				
113	Collagen	E8F4L	250	CST	X
115	HepPar	OCH1E5	250	Abcam	X
141	SMA	1A4	500	Standard BioTools	
142	Podoplanin	D2-40	125	Biolegend	X
143	VIM	D21H3	500	Standard BioTools	
144	CD31	EPR3094	125	Standard BioTools	
145	CD45RO	UCHL1	250	Biolegend	X
146	CD16	EPR16784	100	Standard BioTools	
147	CD163	EDHu-1	125	Standard BioTools	
148	CK (Pan-Keratin)	C11	125	Standard BioTools	
149	CD137	D2Z4Y	250	CST	X
150	PDL1	E1L3N	125	CST	X
151	PD1	D4W2J	125	CST	X
152	CD57	NK/804	250	Standard BioTools	
153	Tox/Tox2	E6I3Q	250	CST	X
154	DC-LAMP	1010E1.01	125	Novus	X
155	FOXP3	PCH101	75	Standard BioTools	

156	CD4	EPR6855	125	Standard BioTools	
158	TTF1	SP141	250	Abcam	X
159	CD68	KP1	75	Standard BioTools	
160	CXCR3	EPR25373-32	250	Abcam	X
161	CD20	H1	125	Standard BioTools	
162	CD8	C8/144B	250	Standard BioTools	
163	ADAM10	Polyclonal	250	Abcam	X
164	ARG1	D4E3M	75	Standard BioTools	
165	CDX2	D11D10	250	CST	X
166	CD45RA	HI100	250	Standard BioTools	
167	GZMB	D6E9W	125	CST	X
168	KI67	B56	250	Standard BioTools	
169	ADAM17	1F6	250	Abcam	X
170	CD3	Polyclonal, C-terminal	125	Standard BioTools	
171	LAG3	17B4	125	Novus	X
158	Tox	REA473	100	Miltenyi Biotec	X
161	CTLA4	14D3	264	Standard BioTools	
171	Gzmb	GB11	66	Standard BioTools	
172	Ki67	B56	66	Standard BioTools	
172	CD15	W6D3	100	Biolegend	X
173	FGL1	EPR24018-27	250	Abcam	X

174	HLADR	LN3	250	Standard BioTools	
175	CD86	E2G8P	125	CST	X
176	CD206	E2L9N	125	CST	X
191	Iridium-DNA				
193	Iridium-DNA				
195	IMC Seg	1A36	250	Standard BioTools	
196	IMC Seg	1A37	250	Standard BioTools	
198	IMC Seg	1A38	250	Standard BioTools	

**Supplemental Table S4. qPCR Primers for interferon response genes**(41–43)	
**Gene Name**	**Sequence**
mMDA5-F	GTGATGACGAGGCCAGCAGTTG
mMDA5-R	ATTCATCCGTTTCGTCCAGTTTCA
mSTAT1-F	GCCTCTCATTGTCACCGAAGAAC
mSTAT1_R	TGGCTGACGTTGGAGATCACCA
mISG15-F	TGACGCAGACTGTAGACACG
mISG15-R	TGGGGCTTTAGGCCATACTC
mMX1-F	GGGGAGGAAATAGAGAAAATGAT
mMX1-R	GTTTACAAAGGGCTTGCTTGCT
mMX2-F	CCAGTTCCTCTCAGTCCCAAGATT
mMX2-R	TACTGGATGATCAAGGGAACGTGG
mIFN-β-F	AAGAGTTACACTGCCTTTGCCATC
mIFN-β-R	CACTGTCTGCTGGTGGAGTTCATC
mcGAS-F	GGCAGCTACTATGAACATGTG
mcGAS-R	CTCAGCGGATTTCCTCGTGGAA
mSTING-F	TATACCTCAGTTGGATGTTTGGC
mSTING-R	CTGGAGTCAAGCTCTGAAGGC
mIL-6-F	TAGTCCTTCCTACCCCAATTTCC
mIL-6-R	TTGGTCCTTAGCCACTCCTTC
mGAPDH-F	TTCCAGTATGACTCCACTCACGG
mGAPDH-R	TGAAGACACCAGTAGACTCCACGAC
mIFNalpha-F	CTCTGTGCTTTCCTGATG
mIFNalpha-R	CCTGAGGTTATGAGTCTGA
mRANTES-F	CAGGAGCAAGTGCTCCAATCTT
mRANTES-R	TTCTTGAACCCACTTCTTCTCTGG

Supplemental Figures

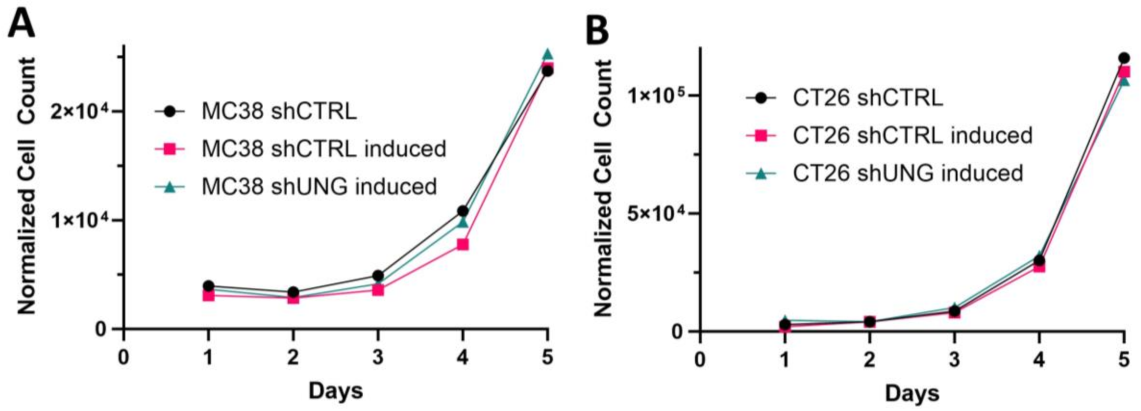

Supplemental Figure S1. Growth rate comparisons of shRNA inducible cell lines. MC38 and CT26 cells with doxycycline inducible shRNA's, shCTRL and shUNG, were cultured alongside uninduced shCTRL cells to compare the growth rate of shUNG knockdowns relative to control cell lines. **(A)** MC38 were plated and cultured and cell count was determined using the MTS assay and normalized to a standard curve of cell dilutions ranging from $1x10^6$ -$1x10^2$ cells. **(B)** CT26 cells were plated and cultured in the same manner as described in panel (A). Normalization involved performing cell dilutions in the range 1 $x10^6$ - 1 $x10^2$ cells.

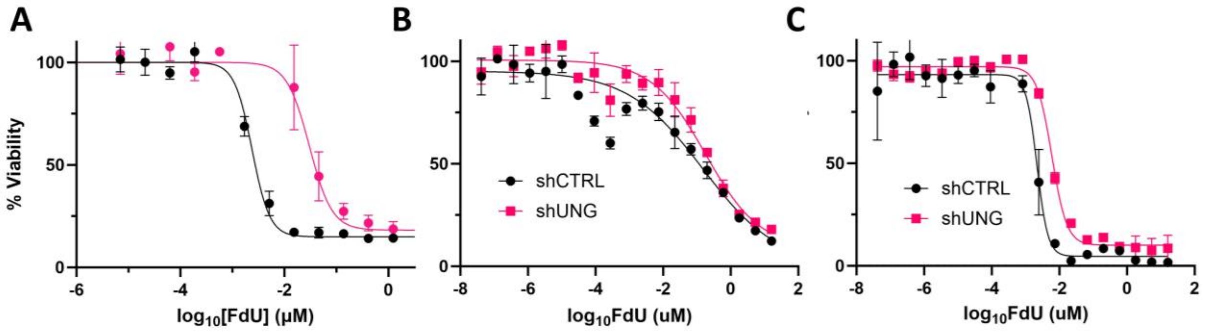

Supplemental Figure S2. Fluorodeoxyuridine (FdU) Dose-Response of shRNA UNG knockdown cell lines in comparison to parental cell lines. (A) MC38 cells containing the cDNA sequence uracil glycosylase inhibitor (UGI) in the CW57.1 doxycycline inducible construct were plated with a half grown in media containing doxycycline (0.5 μg/mL) for 24 hours to induce UGI expression; the other half was grown in Tet-free media. Cells were then plated at 1 x 10^4 per well in a 96 well plate. Mice were treated with FdU for 72 hours and viability was assessed using the MTS reagent relative to untreated controls. **(B)** MC38 cells containing doxycycline inducible shRNA's (shCTRL or shUNG) were plated and exposed to doxycycline followed by treatment with FdU for 72 hours. Viability was assessed using the MTS reagent and normalized to an untreated control. **(C)** CT26 cells with doxycycline inducible shRNA targeting scramble (shCTRL) and UNG (shUNG) were plated and exposed to doxycycline for 24 hours prior to treatment with FdU for 72 hours. Viability was assessed using the MTS reagent and normalized to untreated controls.

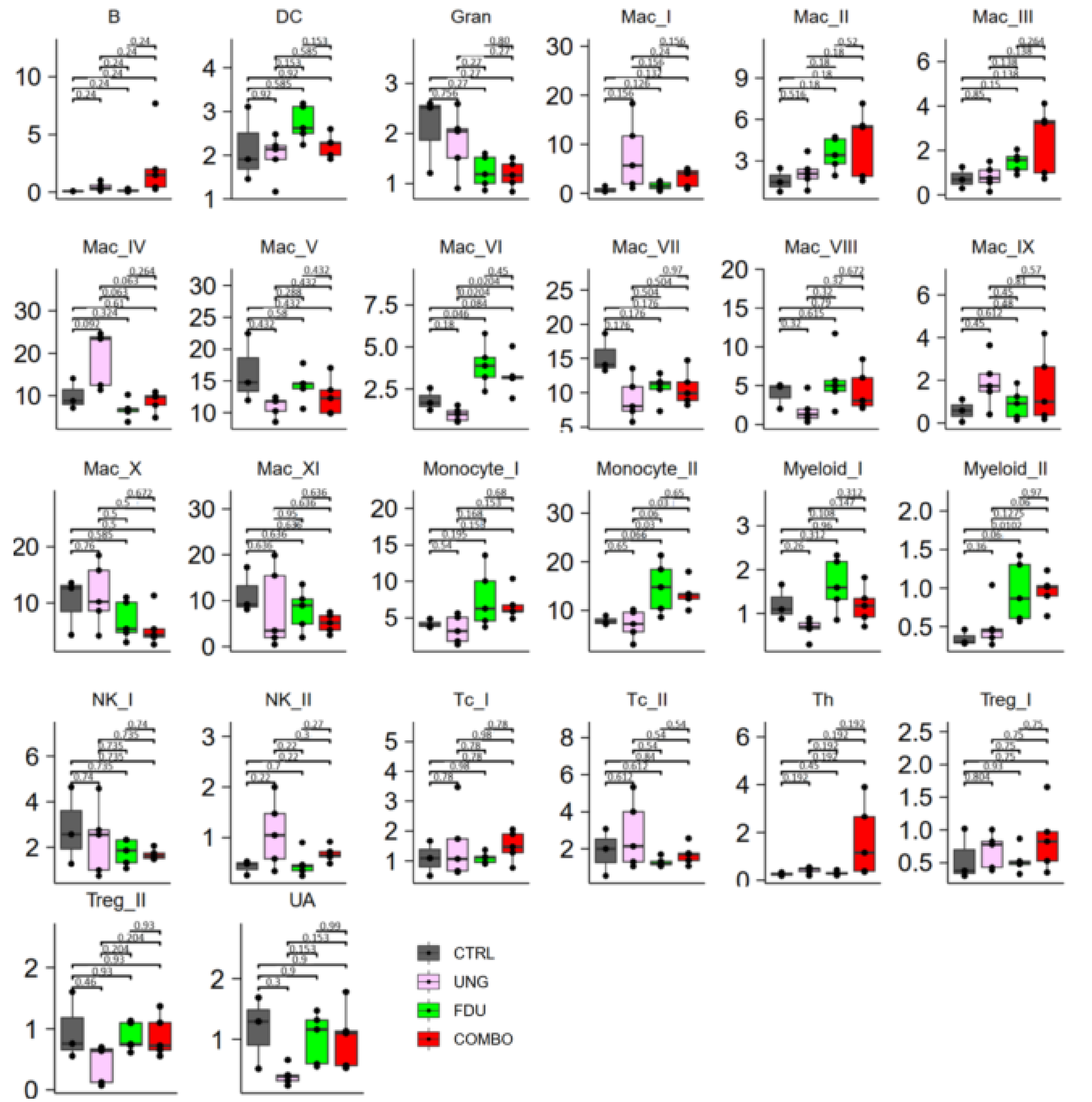

Supplemental Figure S3: Complete list immune cell subpopulation clusters identified within the tumor microenvironment by mass cytometry (CyTOF) following treatment with UGI and/or FdU. This is a complete list of the immune cell subtypes identified as part of this experiment (supplement to Figure 4. To determine the influence of UNG inhibition via UGI on the MC38 tumor microenvironment, 2.5 x 10^5 MC38 cells containing the CW57.1 UGI guide were injected into the right hind limb of female C57/B6 mice. Mice were divided into 4 groups of 10 mice, 1.) Control, 2.) UNG inhibition via UGI induction through with doxycycline, 3.) Floxuridine (FdU) treatment at 50mg/kg/dose daily via intraperitoneal injection days 3 to 12, 4.) UNG inhibition in combination with FdU treatment. On day 20, mice were sacrificed and tumors excised. Tumors were then homogenized and stained with a panel of 40 antibodies tagged with heavy metal isotopes for analysis by mass cytometry (CyTOF). Cell types were clustered based on their expression of cell surface markers. The p-values describing the statistical significance between the two responses are shown for each cell type using 2-tailed T-testing adjusted for multiple comparisons within each cell type using Benjamini-Hochberg (BH). Abbreviations: T_c cells = $CD8^+$ T lymphocytes, T_h cells = $CD4^+$ T lymphocytes, T_{reg} cells = Regulatory T cells, Mac= macrophages, NK cells = natural killer cells, Gran = granulocytes.

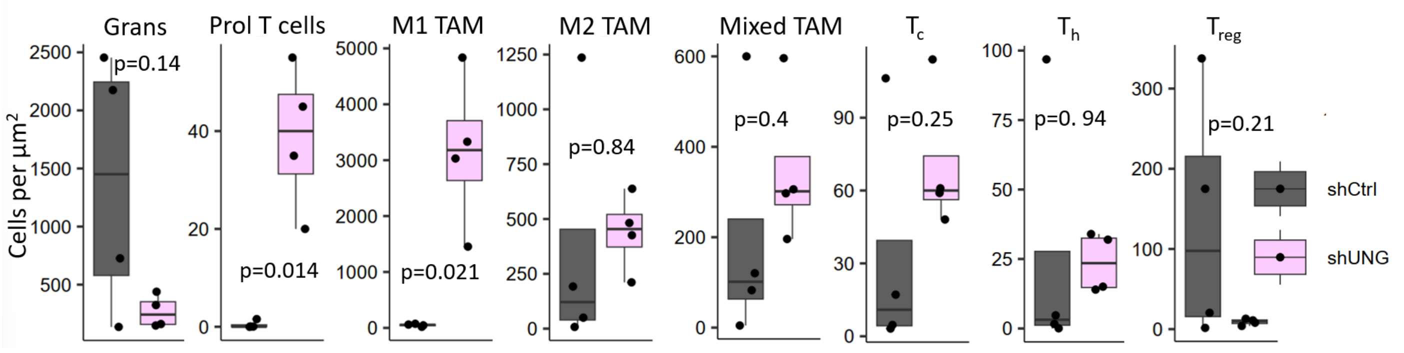

Supplemental Figure S4. Determination of the influence of UNG depletion on the MC38 tumor microenvironment using imaging mass cytometry (IMC). 2.5 x 10^5 MC38 cells expressing $shRNA^{UNG}$ or $shRNA^{ctrl}$ were injected into the right hind limb of female C57/B6 mice (8 total – 4 mice for each experimental group). On day 7, the mice were sacrificed, the tumors excised and fixed in formalin, and then cut and placed onto slides for IMC analysis. The p-values describing the statistical significance between the two responses are shown for each cell type using 2-tailed T-testing. Abbreviations: Proliferative T cells = $CD8^+$, $Ki67^+$, T_c cells = $CD8^+$ T lymphocytes, T_h cells = $CD4^+$ T lymphocytes, Treg cells = $CD4^+$, $FOXP3^+$, Granulocytes = $CD11b^+$, $LY6G^+$, $S100A9^+$, M1 TAM = $CD206^-$, $CD86^+$, $CD68^+$, $KI67^-$ macrophages, M2 TAM = $CD206^+$, $CD68^+$, $Ki67^-$ macrophages, Mixed Subtype TAM = $CD206^{intermediate}$, $CD86^{intermediate}$, $CD68^+$, $Ki67^+$.

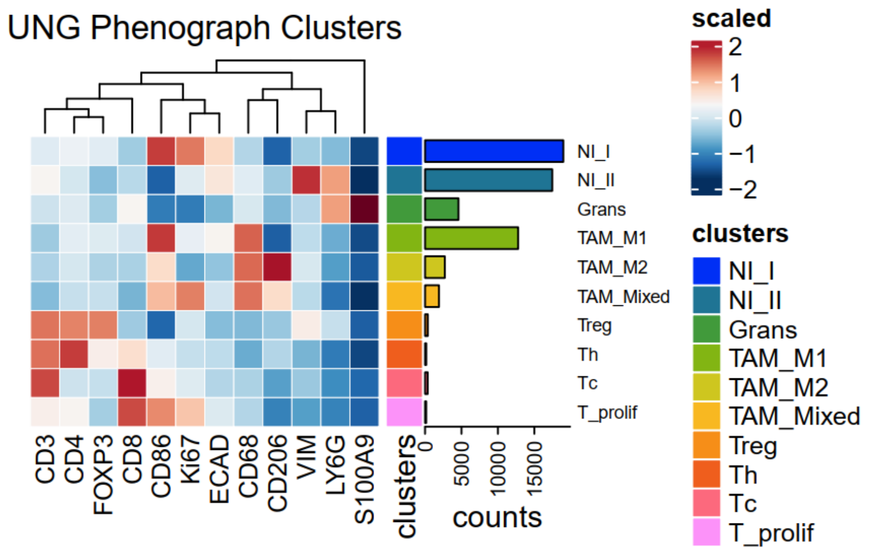

Supplemental Figure S5: Heatmap for tumor microenvironment evaluation with imaging mass cytometry (IMC) of effects of UNG depletion with shRNA on MC38 tumors. To determine the influence of UNG depletion via shRNA on the MC38 tumor microenvironment, 2.5 x 10^5 MC38 cells containing the EZ tet UNG guide or EZ tet control were injected into the right hind limb of female C57/B6 mice (8 total – 4 mice injected with each). On day 7, mice were sacrificed and tumors excised. Tumors were then homogenized and stained with a panel of 40 antibodies tagged with heavy metal isotopes for analysis by mass cytometry (CyTOF). This demonstrated a statistically significant changes in monocyte, dendritic, and macrophage subpopulations as calculated by pairwise t-testing. Cell types were clustered based on their expression of cell surface markers. Abbreviations: T_c cells = $CD8^+$ T lymphocytes, T_h cells = $CD4^+$ T lymphocytes, T_{reg} cells = Regulatory T cells, T_prolif = proliferative T cells, TAM= tumor associated macrophages, NI = not identified subtype, Gran = granulocytes.

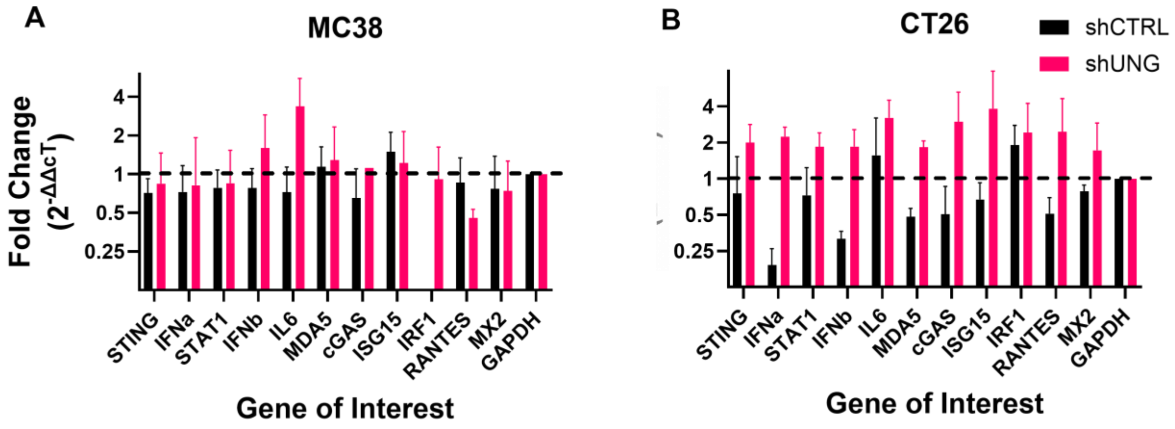

Supplemental Figure S6. Assessment for changes in IFN gene expression upon induction of shRNA targeting UNG. (**A**) Total cellular RNA from MC38 cells was isolated 24 hours after induction of the UNG shRNA and then reversed transcribed prior to transcript quantification by RT-qPCR using previously validated transcript specific primers. (**B**) Total cellular RNA from CT26 cells was isolated and reversed transcribed prior to transcript quantification by RT-qPCR. All data was quantified utilizing ΔΔcT method where ΔΔcT= ΔcT($shRNA^{UNG}$ induced) - ΔcT ($shRNA^{UNG}$ uninduced). All measurements were performed in biological triplicates with three technical replicates for each independent sample. All expression levels are relative to the GAPDH housekeeping gene.

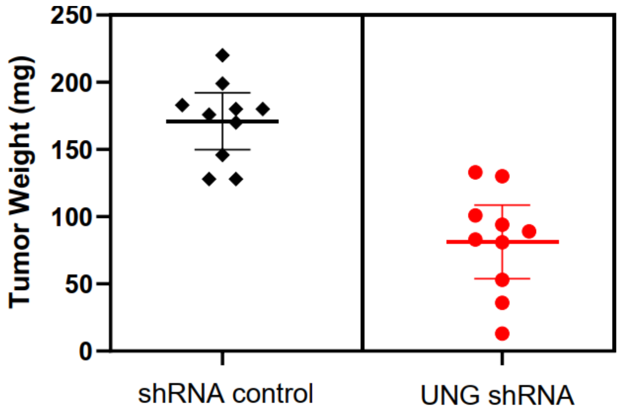

Supplemental Figure S7: MC38 tumor weight after 1 week comparing UNG shRNA knockdown versus control. To determine the influence of UNG depletion via shRNA on the MC38 tumor microenvironment, 2.5 x 10^5 MC38 cells containing the EZ tet UNG guide or EZ tet control were injected into the right hind limb of female C57/B6 mice (20 total – 10 mice injected with each). On day 7, mice were sacrificed and tumors excised. Tumor weights (mg) were determined for each group and plotted. Groups were compared using a 2-tailed student's T-test (p value <0.0001) showing a significantly larger tumor size in mice injected with MC38 tumor cells with shRNA control guide.

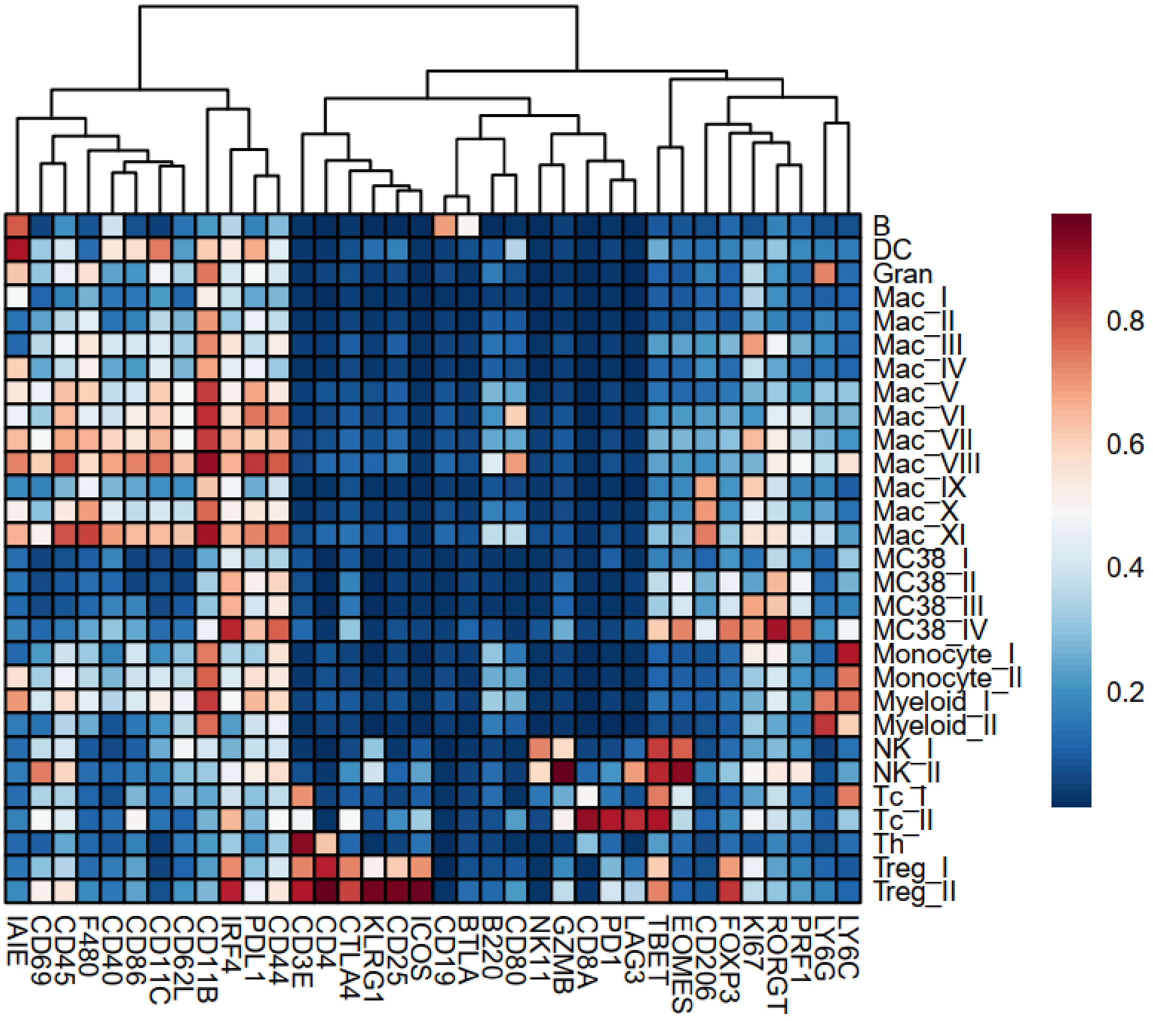

Supplemental Figure S8: Heatmap for tumor microenvironment evaluation with mass cytometry (CyTOF) of effects of UGI in combination with FdU treatment. To determine the influence of UNG inhibition via UGI on the MC38 tumor microenvironment, 2.5 x 10^5 MC38 cells containing the CW57.1 UGI guide were injected into the right hind limb of female C57/B6 mice (20 total). Mice were divided into 4 groups of 10 mice, 1.) Control, 2.) UNG inhibition via UGI induction through with doxycycline starting day 1, 3.) Floxuridine (FdU) treatment at 50mg/kg/dose daily via intraperitoneal injection days 3 to 12, 4.) UNG inhibition in combination with FdU treatment. On day 20, mice were sacrificed and tumors excised. Tumors were then homogenized and stained with a panel of 40 antibodies tagged with heavy metal isotopes for analysis by mass cytometry (CyTOF). Cell types were clustered based on their expression of cell surface markers. Abbreviations: T_c cells = $CD8^+$ T lymphocytes, T_h cells = $CD4^+$ T lymphocytes, T_{reg} cells = Regulatory T cells, Mac= macrophages, NK cells = natural killer cells, Gran = granulocytes.

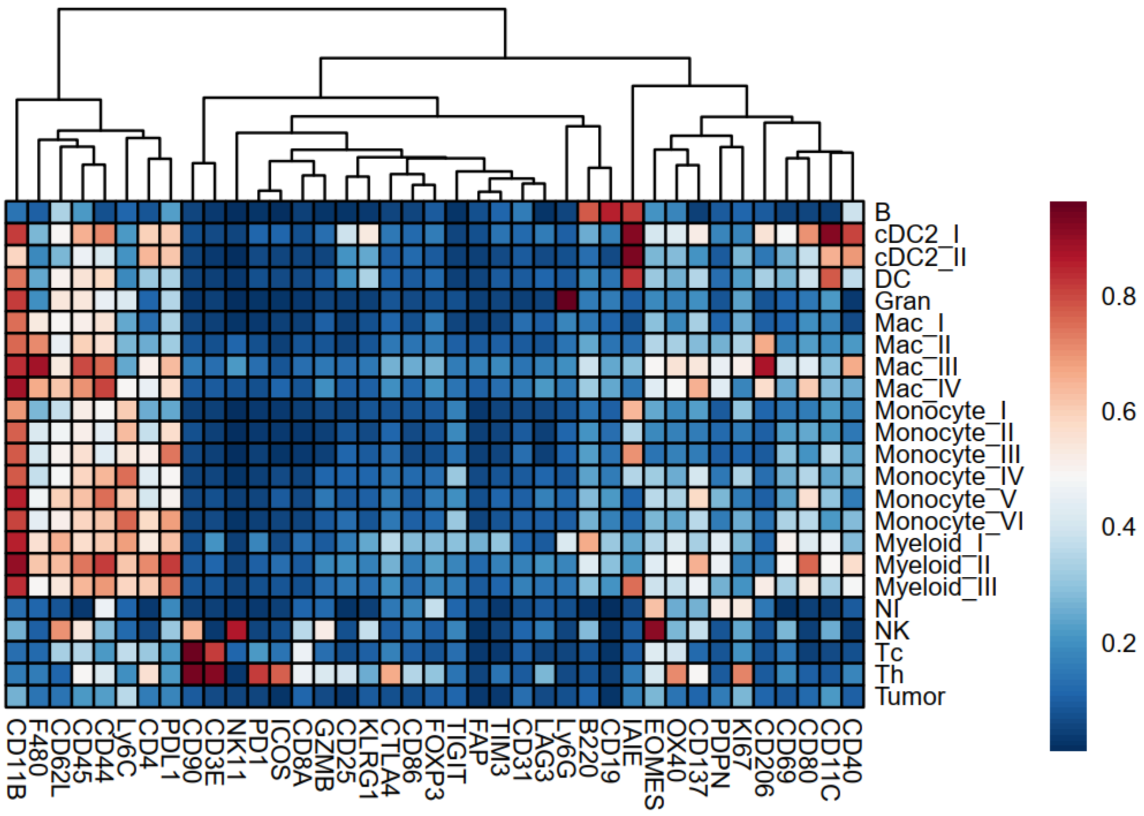

Supplemental Figure S9: Heatmap for tumor microenvironment evaluation with mass cytometry (CyTOF) of effects of UNG depletion. To determine the influence of UNG depletion via shRNA on the MC38 tumor microenvironment, 2.5 x 10^5 MC38 cells containing the EZ tet UNG guide or EZ tet control were injected into the right hind limb of female C57/B6 mice (20 total – 10 mice injected with each). On day 7, mice were sacrificed and tumors excised. Tumors were then homogenized and stained with a panel of 40 antibodies tagged with heavy metal isotopes for analysis by mass cytometry (CyTOF). This demonstrated a statistically significant changes in monocyte, dendritic, and macrophage subpopulations as calculated by Kruskal Wallis testing. Cell types were clustered based on their expression of cell surface markers. Abbreviations: T_c cells = CD8$^+$ T lymphocytes, T_h cells = CD4$^+$ T lymphocytes, T_{reg} cells = Regulatory T cells, Mac= macrophages, NK cells = natural killer cells, Gran = granulocytes.

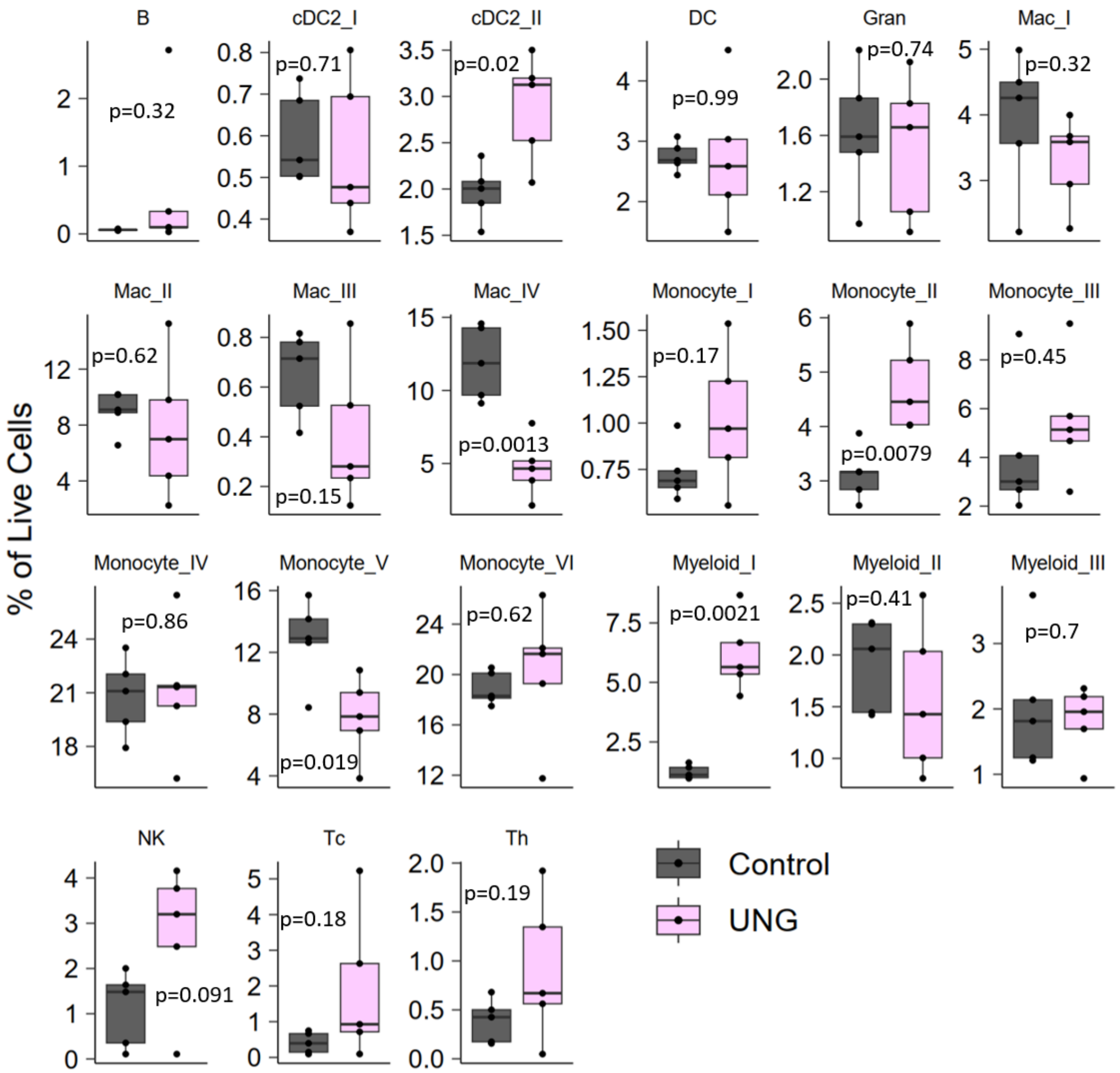

Supplemental Figure S10: Complete list immune cell subpopulation clusters identified within the tumor microenvironment by mass cytometry (CyTOF) following treatment with UNG depletion. This is a complete list of the immune cell subtypes identified as part of this experiment (supplement to Figure 4. To determine the influence of UNG depletion via shRNA on the MC38 tumor microenvironment, 2.5 x 10^5 MC38 cells containing the EZ tet UNG guide or EZ tet control were injected into the right hind limb of female C57/B6 mice (20 total – 10 mice injected with each). On day 7, mice were sacrificed and tumors excised. Tumors were then homogenized and stained with a panel of 40 antibodies tagged with heavy metal isotopes for analysis by mass cytometry (CyTOF). This demonstrated a statistically significant changes in monocyte, dendritic, and macrophage subpopulations as calculated by pairwise T-testing testing. Cell types were clustered based on their expression of cell surface markers. Abbreviations: T_c cells = $CD8^+$ T lymphocytes, T_h cells = $CD4^+$ T lymphocytes, T_{reg} cells = Regulatory T cells, Mac= macrophages, NK cells = natural killer cells, Gran = granulocytes.
