# Supplementary figures and images for: Ablating UNG activity in a mouse model inhibits colorectal cancer growth by increasing tumor immunogenicity
Source: JCI Insight. 2025 Jul 15;10(16):e184435. doi: 10.1172/jci.insight.184435 (PMC12406714; doi:10.1172/jci.insight.184435)

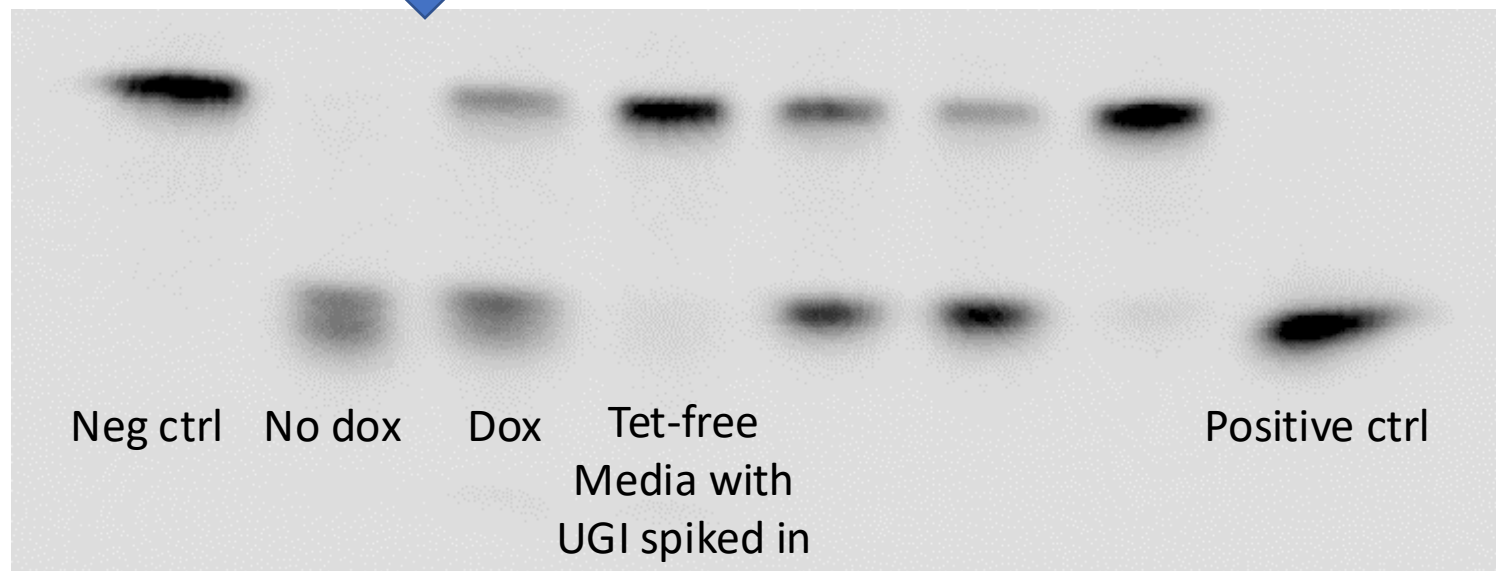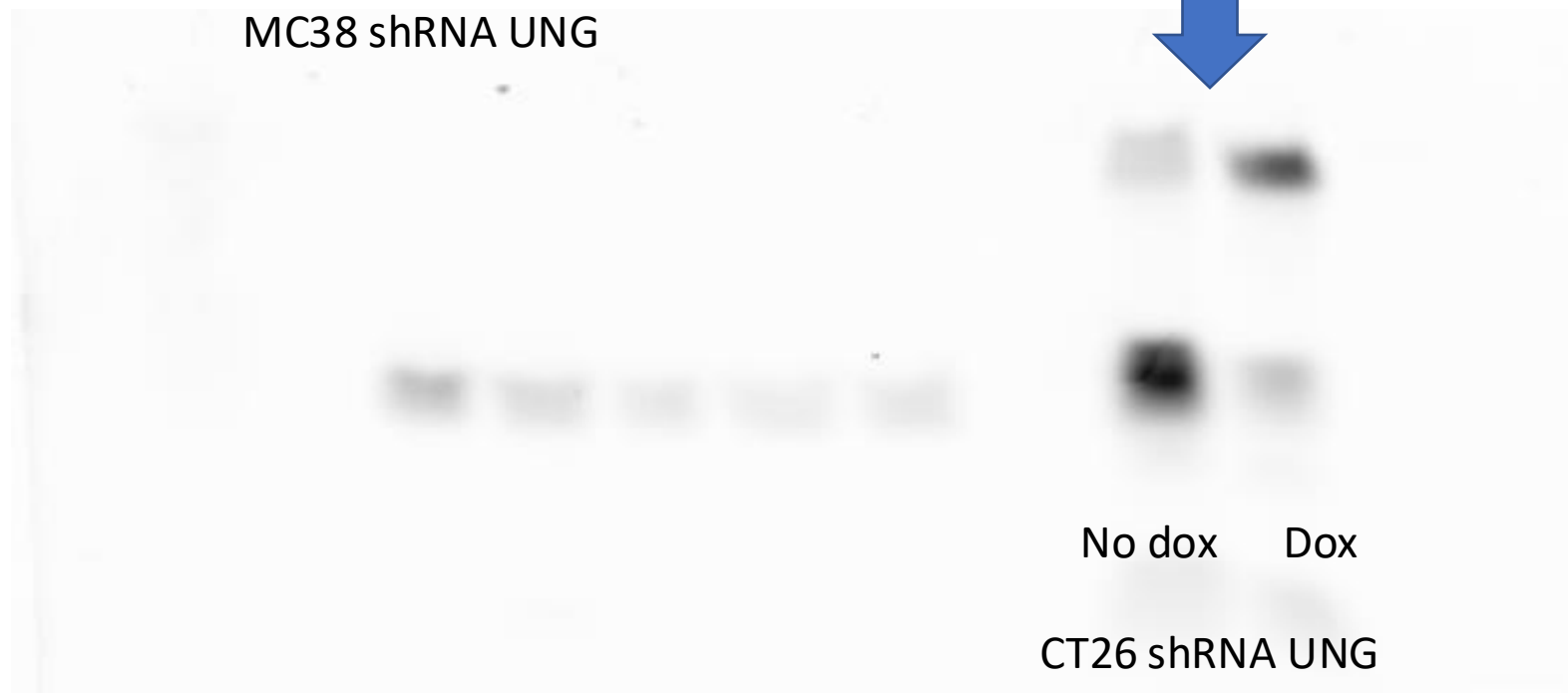

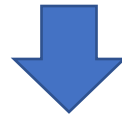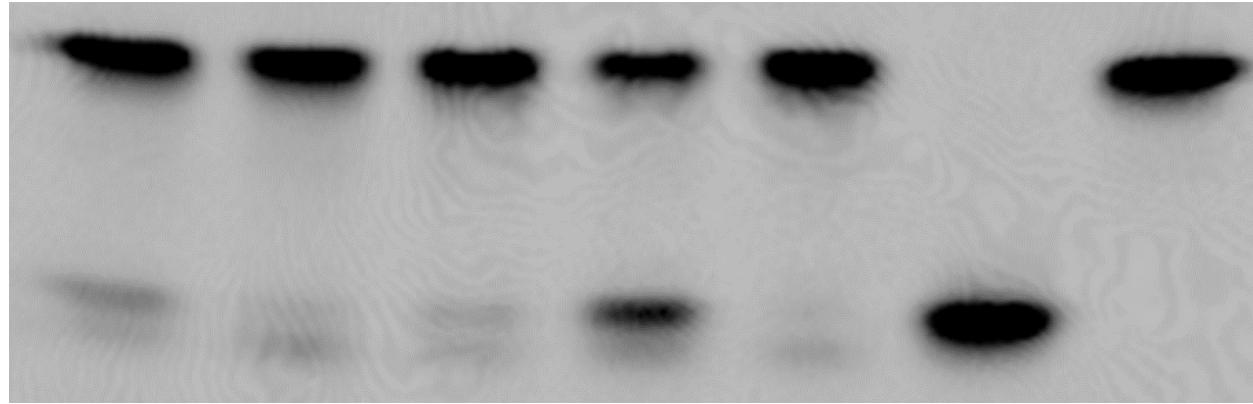

No dox    Dox    Pos ctrl    Neg ctrl

MC38 UGI

Supplement: Unedited blot and gel images [file jciinsight-10-184435-s100.pdf]
